# Contact-Reliability-Aware Neural Residual Kalman Filtering for Legged Robot State Estimation

**DOI:** 10.3390/s26196041

**Published:** 2026-09-23

**Authors:** Jiawei Zhao, Yuping Huang, Ke Li, Jingxuan Cao, Yinan Liu, Yanjiang Chen, Linfan Yu

**Affiliations:** 1 Beijing Institute of Precision Mechatronics and Controls, No.1 South DaHongMen Road, Fengtai District, Beijing 100076, China; zhaojiawei93@163.com (J.Z.); keli@stu.hit.edu.cn (K.L.); caojx2024@163.com (J.C.); liuyinan2021@163.com (Y.L.); cyj_cvrobot@163.com (Y.C.); yulf@zju.edu.cn (L.Y.); 2 Innovation Center for Control Actuators, No.1 South DaHongMen Road, Fengtai District, Beijing 100076, China

**Keywords:** legged robot state estimation, proprioceptive sensing, contact-aided state estimation, continuous contact reliability, Kalman filtering, neural residual learning

## Abstract

In legged robot state estimation without foot-contact measurements, nominal gait phase does not directly indicate the instantaneous validity of foot–ground kinematic constraints, while residual errors in state propagation and kinematic pseudo-measurements can further bias the state estimate. A contact-reliability-aware neural residual Kalman filtering framework is developed while retaining the physics-based state-space model and Kalman recursion. A multi-task temporal estimator infers gait-phase-constrained continuous contact reliability together with dynamic and observation residuals from proprioceptive history sequences. The estimated reliability regulates contact-related process and measurement covariances to adjust the confidence assigned to foot–ground kinematic constraints. The dynamic residual compensates base-velocity prediction errors, and the observation residual corrects predictable errors in foot kinematic pseudo-measurements through the measurement innovation. Simulation results show lower base-state and foot-position errors than the Binary Contact Kalman filter across multiple motion commands. Physical walking and turning experiments show improved horizontal base trajectory estimation and lower terminal drift within the prescribed onboard update period.

## 1. Introduction

Legged robot state estimation commonly combines inertial measurement unit (IMU) measurements with leg kinematics derived from joint encoders to estimate the base position and linear velocity [1,2]. Contact-aided estimators further use the approximately stationary support foot as a kinematic reference to constrain the drift accumulated from inertial integration [3,4,5]. The effectiveness of this constraint depends on whether the foot remains sufficiently stationary relative to the ground. During locomotion, contact transitions and non-ideal contact conditions can reduce the validity of the foot–ground kinematic constraint [6]. In gait-phase-conditioned locomotion, the nominal gait phase specifies the intended contact timing but does not directly indicate the instantaneous validity of the foot–ground kinematic constraint [7]. When the realized contact timing or condition deviates from the nominal gait schedule [8], predefined contact rules may assign inappropriate confidence to the corresponding foot–ground kinematic constraints, leading to accumulated estimation errors.

Recursive state estimation for legged robots has been implemented using both Kalman filtering and particle filtering (PF) [9]. PF represents the posterior distribution with weighted particles and can handle nonlinear and non-Gaussian uncertainty, but its computational cost increases with the number of particles [10]. Given the linear state-space structure of the estimation problem considered here and the need for efficient onboard computation, Kalman filtering is adopted. Existing Kalman-filter-based estimators handle unreliable foot constraints mainly by changing the contact representation or the uncertainty assigned to foot kinematic measurements. Binary contact-aided filters activate or deactivate foot constraints according to discrete contact states, which limits their ability to represent gradual changes around touchdown and liftoff [11]. Multiple-model Kalman filters extend this idea by treating different contact combinations as discrete modes and jointly estimating the contact mode and robot state [12]. Other methods estimate contact probabilities from proprioceptive or contact-related measurements and use them to adjust the influence of the corresponding foot constraints [13,14]. Contact-event information has also been incorporated into recursive estimators to improve state estimation during contact transitions [15,16]. A related line of work regulates contact uncertainty through filter covariances. Adaptive estimators adjust covariance terms using online noise or contact information [17,18,19]. CoCo-InEKF, based on a differentiable invariant extended Kalman filter (InEKF), further learns continuous contact velocity covariances from proprioceptive histories [20]. These methods represent contact uncertainty using discrete states, contact probabilities, or adaptive covariances. However, they generally do not use the temporal structure of the nominal gait phase to constrain the evolution of contact confidence. Around touchdown and liftoff, transient motion and contact changes can cause local fluctuations or timing variations in the estimated confidence, leading to a mismatch between contact-related uncertainty regulation and the actual validity of the foot–ground kinematic constraint.

Without foot-contact measurements, foot–ground kinematic constraint validity must be inferred from proprioception. Temporal patterns in IMU and joint signals provide motion- and contact-related information beyond individual observations. Proprioceptive histories have been applied to learning-based state estimation and leg-inertial odometry [21,22,23], locomotion phase estimation [24], and contact inference within invariant filtering frameworks [25,26]. Contact-related covariance regulation changes the confidence assigned to prediction or measurement information without altering the nominal state propagation or pseudo-measurement models. It can reduce the influence of unreliable constraints but does not directly correct predictable errors in the predicted state or foot kinematic pseudo-measurements. Learned relative motion and uncertainty have been incorporated into extended Kalman filter (EKF) updates [27], while proprioceptive histories have been used to estimate yaw information for multi-IMU state estimation [28]. InEKFormer uses a Transformer to estimate the Kalman gain from temporal information while retaining the state propagation and correction structure of the InEKF [29]. Neural corrections have also been introduced to compensate for model mismatch, contact bias, and slip-related errors in Kalman filtering frameworks [30,31,32]. These studies show that temporal proprioceptive information can be used to infer motion quantities, contact information, or filter parameters and improve recursive state estimation. However, the reviewed methods mainly augment individual components of the filter and do not explicitly separate prediction-stage errors from foot kinematic pseudo-measurement errors for joint compensation within the same Kalman recursion.

This paper presents a contact-reliability-aware neural residual Kalman filtering framework for legged robot state estimation. The method retains the physics-based state-space model and Kalman recursion, while introducing gait-phase-constrained continuous contact reliability together with residual corrections at the prediction and measurement-update stages. The contact reliability represents the estimated validity of the foot–ground kinematic constraint throughout the gait cycle, including its continuous variations around normal touchdown and liftoff transitions, and regulates the corresponding process and measurement covariances. The dynamic and observation residuals compensate the prior base-velocity error and the foot kinematic pseudo-measurement error, respectively. The main contributions are as follows:

1. A contact-reliability-aware neural residual Kalman filtering framework retains the physics-based state-space model and recursive Kalman structure while incorporating continuous contact reliability and stage-specific residual corrections.
2. A gait-phase-constrained continuous contact reliability model uses nominal gait timing as a structural prior and allows bounded adjustment of gait-phase and contact-transition parameters, providing continuous reliability for contact-related covariance regulation.
3. A stage-specific residual correction scheme separately compensates base-velocity prediction errors and foot kinematic pseudo-measurement errors through dynamic and observation residuals at the prediction and measurement-update stages, respectively.

## 2. Materials and Methods

This section presents the proposed contact-reliability-aware neural residual Kalman filtering framework. The following subsections describe the overall architecture, multi-task temporal estimator, gait-phase-constrained continuous contact reliability generation, contact-reliability-aware neural residual Kalman filtering, and the privileged supervision and training objective. For reference, the principal symbols and notation used throughout the manuscript are summarized in Table A1 of Appendix A.

### 2.1. Overview of the Proposed Framework

The proposed contact-reliability-aware neural residual Kalman filtering framework consists of three main components: a proprioceptive sequence construction module, a multi-task temporal estimator, and a contact-reliability-aware neural residual Kalman filter, as shown in Figure 1. The proprioceptive history sequence is encoded by the temporal estimator to generate contact-related latent variables, dynamic residuals, and observation residuals. The contact-related latent variables are combined with the nominal gait phase to generate continuous contact reliability for process and measurement covariance regulation, while the dynamic and observation residuals correct the prior base velocity and measurement innovation, respectively.

### 2.2. Multi-Task Temporal Estimator

At time step *t*, the input vector is defined as

(1) $$o_{t}={\left[{\left(a_{m,t}^{B}\right)}^{T},{\left(\omega_{m,t}^{B}\right)}^{T},q_{t}^{T},{\dot{q}}_{t}^{T},{\left(u_{t}^{\mathrm{cmd}}\right)}^{T},\phi_{t}^{T}\right]}^{T}\in R^{d_{o}},$$

where $d_{o}=2n_{j}+11$ is the input dimension, and $n_{j}$ denotes the number of actuated joints. The vectors $a_{m,t}^{B},\omega_{m,t}^{B}\in R^{3}$ denote the IMU specific-force and angular-velocity measurements, respectively, while $q_{t},{\dot{q}}_{t}\in R^{n_{j}}$ denote the joint-position and joint-velocity measurements from the encoders. The motion command vector $u_{t}^{\mathrm{cmd}}={\left[v_{x,t}^{\mathrm{cmd}},v_{y,t}^{\mathrm{cmd}},\omega_{z,t}^{\mathrm{cmd}}\right]}^{T}\in R^{3}$ contains the commanded forward and lateral velocities and yaw angular velocity. The gait phase vector $\phi_{t}={\left[\phi_{L,t},\phi_{R,t}\right]}^{T}\in R^{2}$ contains the nominal gait phases of the left and right feet defined by the reference gait of the reinforcement-learning locomotion policy.

An input history of length *T* is constructed as $O_{t-T+1:t}={\left[o_{t-T+1},\ldots ,o_{t}\right]}^{T}\in R^{T\times d_{o}}$ and normalized feature-wise to obtain ${\bar{O}}_{t-T+1:t}$. A fast Fourier transform (FFT) is then applied along the temporal dimension, from which the magnitude feature $M_{t}=\left|{\mathrm{FFT}}_{T}\left({\bar{O}}_{t-T+1:t}\right)\right|$ and phase feature $\psi_{t}=∠{\mathrm{FFT}}_{T}\left({\bar{O}}_{t-T+1:t}\right)$ are extracted. The normalized time-domain sequence, magnitude spectrum, and phase spectrum are concatenated along the feature dimension to form $\left[{\bar{O}}_{t-T+1:t},M_{t},\psi_{t}\right]\in R^{T\times 3d_{o}}$. The time-domain sequence serves as the primary temporal representation, while the Fourier magnitude and phase provide auxiliary descriptors of gait periodicity and contact transitions [33].

The concatenated representation is mapped to the hidden space through an input embedding layer and combined with positional encoding. The resulting sequence is processed by stacked Transformer encoder blocks with multi-head self-attention, feed-forward networks, residual connections, and layer normalization [34]. The encoded sequence is mean-pooled along the sequence dimension to obtain a shared temporal feature, which is then passed to three task-specific multilayer perceptron (MLP) heads, yielding

(2) $$\left(z_{t}^{c},r_{t}^{\mathrm{dyn}},r_{t}^{\mathrm{obs}}\right)=f_{\theta }\left(O_{t-T+1:t}\right),$$

where $f_{\theta }\left(\cdot \right)$ denotes the multi-task temporal estimator parameterized by θ, comprising input normalization, auxiliary Fourier descriptor construction, Transformer encoding, temporal mean pooling, and three task-specific MLP heads. The output $z_{t}^{c}\in R^{8}$ denotes the contact-related latent representation of the two feet, $r_{t}^{\mathrm{dyn}}\in R^{3}$ denotes the dynamic residual in the base velocity space, and $r_{t}^{\mathrm{obs}}\in R^{14}$ denotes the observation residual in the pseudo-measurement space.

### 2.3. Gait-Phase-Constrained Continuous Contact Reliability Generation

The nominal gait phase is combined with the contact-related representation from the multi-task temporal estimator to generate continuous contact reliability. For foot i∈{L,R}, the corresponding contact-related latent vector is defined as

(3) $$z_{i,t}^{c}={\left[z_{i,t}^{\varphi },z_{i,t}^{\alpha },z_{i,t}^{u},z_{i,t}^{d}\right]}^{T}\in R^{4}.$$

Here, $z_{i,t}^{\varphi }$ adjusts the contact phase correction, $z_{i,t}^{\alpha }$ adjusts the swing phase ratio, and $z_{i,t}^{u}$ and $z_{i,t}^{d}$ adjust the touchdown and liftoff transition widths, respectively. The latent vectors of the two feet are concatenated as $z_{t}^{c}={\left[{\left(z_{L,t}^{c}\right)}^{T},{\left(z_{R,t}^{c}\right)}^{T}\right]}^{T}\in R^{8}$. The reliability generation process is illustrated in Figure 2.

Based on the contact-related latent vector of each foot, the phase correction, corrected phase, swing phase ratio, and touchdown and liftoff transition widths are defined as

(4) $$\left\{\begin{array}{cc} \Delta \varphi_{i,t} & =\Delta \varphi_{i}^{0}+\Delta \varphi_{\max}\tanh\;\left(z_{i,t}^{\varphi }\right),\\ {\tilde{\phi }}_{i,t} & =\mathrm{mod}\;\left(\phi_{i,t}+\Delta \varphi_{i,t},1\right),\\ \alpha_{i,t} & =\mathrm{clip}\;\left(\alpha_{i}^{0}+\Delta \alpha_{\max}\tanh\;\left(z_{i,t}^{\alpha }\right),\alpha_{\min},\alpha_{\max}\right),\\ \epsilon_{i,t}^{u} & =\epsilon_{\min}+\epsilon_{\mathrm{range}}\sigma \;\left(z_{i,t}^{u}\right),\\ \epsilon_{i,t}^{d} & =\epsilon_{\min}+\epsilon_{\mathrm{range}}\sigma \;\left(z_{i,t}^{d}\right). \end{array}\right.$$

Here, $\Delta \varphi_{i}^{0}$ and $\Delta \varphi_{\max}>0$ denote the fixed phase bias and the maximum learned adjustment, respectively. $\phi_{i,t}\in \left[0,1\right)$ and ${\tilde{\phi }}_{i,t}\in \left[0,1\right)$ denote the nominal and corrected gait phases of foot *i*, respectively. $\alpha_{i}^{0}$ and $\Delta \alpha_{\max}>0$ denote the nominal swing phase ratio and its maximum adjustment, while $\alpha_{\min}$ and $\alpha_{\max}$ specify the admissible range of $\alpha_{i,t}$. The tanh(·) function maps $z_{i,t}^{\varphi }$ and $z_{i,t}^{\alpha }$ to signed bounded factors for adjusting the phase correction and swing phase ratio, respectively, limiting excessive phase drift and swing-ratio variation. The corresponding stance phase ratio is $\rho_{i,t}=1-\alpha_{i,t}$. $\epsilon_{\min}>0$ and $\epsilon_{\mathrm{range}}>0$ define the initial lower bound and adjustable range of the touchdown and liftoff transition widths, while σ(·) denotes the sigmoid function and bounds the corresponding width adjustments to (0,1). The resulting $\epsilon_{i,t}^{u}$ and $\epsilon_{i,t}^{d}$ determine the durations of the touchdown and liftoff transition intervals, respectively. To prevent excessively broad transitions, the widths are further constrained as $\epsilon_{i,t}^{u}\leftarrow \min\left\{\epsilon_{i,t}^{u},\beta \rho_{i,t}\right\}$ and $\epsilon_{i,t}^{d}\leftarrow \min\left\{\epsilon_{i,t}^{d},\beta \rho_{i,t},\beta \left(1-\rho_{i,t}\right)\right\}$, where 0<β≤2/3 is a fixed transition-width constraint coefficient. These constraints prevent overlap between the touchdown and liftoff transition intervals and preserve the temporal ordering of touchdown, stable stance, liftoff, and swing. The operators clip(·) and mod(·,1) enforce the admissible range of the swing phase ratio and gait phase periodicity, respectively.

Based on the corrected phase ${\tilde{\phi }}_{i,t}$, the stance phase ratio $\rho_{i,t}$, and the transition widths $\epsilon_{i,t}^{u}$ and $\epsilon_{i,t}^{d}$, the touchdown rising term and liftoff falling term are defined as

(5) $$\left\{\begin{array}{cc} s_{i,t}^{u}\;\left({\tilde{\phi }}_{i,t}\right) & =\sigma \;\left[\gamma \frac{{\tilde{\phi }}_{i,t}-0.5\epsilon_{i,t}^{u}}{\epsilon_{i,t}^{u}}\right],\\ s_{i,t}^{d}\;\left({\tilde{\phi }}_{i,t}\right) & =\sigma \;\left[\gamma \frac{\rho_{i,t}-{\tilde{\phi }}_{i,t}}{\epsilon_{i,t}^{d}}\right]. \end{array}\right.$$

The corrected phase is referenced such that ${\tilde{\phi }}_{i,t}=0$ marks the onset of the touchdown transition. The term $s_{i,t}^{u}$ increases smoothly during the touchdown transition, whereas $s_{i,t}^{d}$ decreases smoothly during the liftoff transition. The fixed coefficient γ>0 controls the transition steepness.

The continuous contact reliability of foot *i* is defined as

(6) $$c_{i,t}\;\left({\tilde{\phi }}_{i,t}\right)=s_{i,t}^{u}\;\left({\tilde{\phi }}_{i,t}\right)s_{i,t}^{d}\;\left({\tilde{\phi }}_{i,t}\right).$$

The multiplicative form yields high reliability after the touchdown transition and before the liftoff transition. Accordingly, $c_{i,t}$ remains close to one during stable stance, approaches zero during swing, and varies smoothly during contact transitions. The resulting $c_{i,t}$ represents the continuous reliability of the corresponding foot–ground kinematic constraint rather than a calibrated probability of physical contact.

Before covariance regulation, $c_{i,t}$ is clipped to $\left[c_{\min},c_{\max}\right]$ to avoid assigning complete trust or complete rejection to the corresponding foot–ground kinematic constraint, where $c_{\min}$ and $c_{\max}$ denote the lower and upper reliability bounds, respectively. For simplicity, $c_{i,t}$ denotes the clipped reliability in the subsequent filtering equations. The reliabilities of the two feet are then collected as $c_{t}={\left[c_{L,t},c_{R,t}\right]}^{T}\in R^{2}$.

### 2.4. Contact-Reliability-Aware Neural Residual Kalman Filter

This subsection describes how the estimated contact reliability, dynamic residual, and observation residual are incorporated into the Kalman filtering recursion. The state-space and contact-aided measurement models are first formulated, followed by reliability-aware covariance regulation, dynamic residual correction in prediction, and observation residual correction in the measurement update.

#### 2.4.1. State-Space Model and Contact-Aided Measurement Model

Let {B} and {W} denote the robot body frame and inertial world frame, respectively. The base orientation $R_{B,t}^{W}\in \mathrm{SO}\left(3\right)$ maps vectors from {B} to {W} and is provided by an IMU-based attitude estimator. The filter state is defined as

(7) $$x_{t}={\left[{\left(p_{b,t}^{W}\right)}^{T},{\left(v_{b,t}^{W}\right)}^{T},{\left(p_{L,t}^{W}\right)}^{T},{\left(p_{R,t}^{W}\right)}^{T},b_{a,t}^{T}\right]}^{T}\in R^{15},$$

where $p_{b,t}^{W}$ and $v_{b,t}^{W}$ are the base position and linear velocity, $p_{L,t}^{W}$ and $p_{R,t}^{W}$ are the left and right foot positions, and $b_{a,t}$ is an auxiliary acceleration compensation state accounting for slowly varying acceleration-related propagation errors. All components belong to $R^{3}$ and are expressed in {W}.

The translational acceleration used for propagation is $a_{b,t-1}^{W}=R_{B,t-1}^{W}a_{m,t-1}^{B}+g^{W}$, where $a_{m,t-1}^{B}$ is the accelerometer specific-force measurement in {B}, and $g^{W}$ is the gravitational acceleration in {W}. Using first-order discretization, the state model is

(8) $$\begin{array}{cc} x_{t} & =Ax_{t-1}+Ba_{b,t-1}^{W}+w_{t-1},\\ A & =\left[\begin{array}{ccccc} I_{3} & \Delta tI_{3} & 0_{3} & 0_{3} & 0_{3}\\ 0_{3} & I_{3} & 0_{3} & 0_{3} & \Delta tI_{3}\\ 0_{3} & 0_{3} & I_{3} & 0_{3} & 0_{3}\\ 0_{3} & 0_{3} & 0_{3} & I_{3} & 0_{3}\\ 0_{3} & 0_{3} & 0_{3} & 0_{3} & I_{3} \end{array}\right],\;\;\;B={\left[\begin{array}{ccccc} 0_{3} & \Delta tI_{3} & 0_{3} & 0_{3} & 0_{3} \end{array}\right]}^{T}. \end{array}$$

Here, $I_{3}$ and $0_{3}$ denote the 3×3 identity and zero matrices, respectively, and Δt is the filter sampling period. $w_{t-1}\in R^{15}$ denotes the zero-mean process disturbance with covariance $Q_{t-1}\in R^{15\times 15}$. The left and right foot positions and the acceleration compensation state follow random-walk models.

For foot i∈{L,R}, the foot position and velocity relative to the base, expressed in {B}, are obtained as $p_{i,t}^{B}={\mathrm{FK}}_{i}\left(q_{t}\right)$ and $v_{i,t}^{B}=J_{i}\left(q_{t}\right){\dot{q}}_{t}$, where ${\mathrm{FK}}_{i}\left(\cdot \right)$ and $J_{i}\left(\cdot \right)$ are the forward kinematics and translational Jacobian, respectively, while $q_{t}$ and ${\dot{q}}_{t}$ are the joint positions and velocities. The corresponding foot position and velocity in {W} satisfy $p_{i,t}^{W}=p_{b,t}^{W}+R_{B,t}^{W}p_{i,t}^{B}$ and $v_{i,t}^{W}=v_{b,t}^{W}+R_{B,t}^{W}\left(\omega_{t}^{B}\times p_{i,t}^{B}+v_{i,t}^{B}\right)$, where $\omega_{t}^{B}$ is the filtered base angular velocity obtained from the IMU.

Using these kinematic relations, the stationary contact condition $v_{i,t}^{W}\approx 0$, and the local ground height $h_{i,t}^{g}$, the pseudo-measurements are constructed as

(9) $$\left\{\begin{array}{cc} y_{i,t}^{p} & =-R_{B,t}^{W}p_{i,t}^{B},\\ y_{i,t}^{v} & =R_{B,t}^{W}\left(\omega_{t}^{B}\times p_{i,t}^{B}+v_{i,t}^{B}\right),\\ y_{i,t}^{h} & =h_{i,t}^{g}. \end{array}\right.$$

The complete measurement vector is

(10) $$y_{t}={\left[{\left(y_{L,t}^{p}\right)}^{T},{\left(y_{R,t}^{p}\right)}^{T},{\left(y_{L,t}^{v}\right)}^{T},{\left(y_{R,t}^{v}\right)}^{T},y_{L,t}^{h},y_{R,t}^{h}\right]}^{T}\in R^{14}.$$

Accordingly, the corresponding measurement model is written as

(11) $$\begin{array}{cc} y_{t} & =Cx_{t}+\nu_{t},\\ C & =\left[\begin{array}{ccccc} I_{3} & 0_{3} & -I_{3} & 0_{3} & 0_{3}\\ I_{3} & 0_{3} & 0_{3} & -I_{3} & 0_{3}\\ 0_{3} & -I_{3} & 0_{3} & 0_{3} & 0_{3}\\ 0_{3} & -I_{3} & 0_{3} & 0_{3} & 0_{3}\\ 0_{1\times 3} & 0_{1\times 3} & e_{z}^{T} & 0_{1\times 3} & 0_{1\times 3}\\ 0_{1\times 3} & 0_{1\times 3} & 0_{1\times 3} & e_{z}^{T} & 0_{1\times 3} \end{array}\right]\in R^{14\times 15}. \end{array}$$

Here, $\nu_{t}\in R^{14}$ denotes the pseudo-measurement error, and $e_{z}={\left[0,0,1\right]}^{T}$ is the vertical unit vector. The position pseudo-measurement represents $p_{b,t}^{W}-p_{i,t}^{W}$, the velocity pseudo-measurement represents $-v_{b,t}^{W}$ under the stationary contact assumption, and the height pseudo-measurement constrains $e_{z}^{T}p_{i,t}^{W}$ to $h_{i,t}^{g}$. At filter initialization, $h_{i,t}^{g}$ is computed from the current base position estimate and foot forward kinematics. When the foot enters a stable stance phase, the reference height is reset using the latest available estimate and is subsequently held constant throughout the corresponding stance interval.

Although the kinematic and coordinate transformations involve nonlinear operations, the measured joint states, angular velocity, and base orientation are not included in the filter state and are available before the Kalman recursion. The IMU specific force is also transformed into the world frame before state propagation. Therefore, (8) and (11) form a linear state-space model with respect to the selected filter state, providing the basis for the linear Kalman filter recursion used in the proposed estimator.

#### 2.4.2. Reliability-Aware Covariance Regulation

The continuous contact reliability $c_{i,t}$ of foot i∈{L,R}, defined in Section 2.3, regulates the corresponding process and measurement uncertainties. Specifically, $c_{i,t-1}$ modulates the process uncertainty of the corresponding foot position during prediction, whereas $c_{i,t}$ modulates the confidence assigned to the associated pseudo-measurements during the measurement update. Consistent with the state ordering in (7), the process covariance is defined as

(12) $$\left\{\begin{array}{cc} Q_{t-1} & =\mathrm{blkdiag}\;\left(Q_{p},Q_{v},Q_{L,t-1}^{f},Q_{R,t-1}^{f},Q_{b_{a}}\right)+BQ_{a}B^{T},\\ Q_{i,t-1}^{f} & =\lambda_{i,t-1}Q_{i,0}^{f},\\ \lambda_{i,t-1} & =c_{i,t-1}+\left(1-c_{i,t-1}\right)T_{R}. \end{array}\right.$$

Here, blkdiag(·) denotes block-diagonal matrix construction, and $Q_{t-1}\in R^{15\times 15}$ is the process covariance. The matrices $Q_{p}$, $Q_{v}$, $Q_{b_{a}}$, and $Q_{a}$ are all in $R^{3\times 3}$ and specify the base position, base velocity, acceleration-compensation, and acceleration-input covariances, respectively. $Q_{i,0}^{f}\in R^{3\times 3}$ is the reference foot position process covariance, and $T_{R}>1$ is the covariance amplification factor. Thus, a lower $c_{i,t-1}$ increases the process uncertainty of the corresponding foot position.

Following the pseudo-measurement ordering in (10), the contact-reliability matrix is defined as

(13) $$\Gamma_{t}=\mathrm{blkdiag}\;\left(c_{L,t}I_{3},c_{R,t}I_{3},c_{L,t}I_{3},c_{R,t}I_{3},c_{L,t},c_{R,t}\right).$$

Here, $\Gamma_{t}\in R^{14\times 14}$. The six diagonal blocks correspond sequentially to the position pseudo-measurements of the left and right feet, the velocity pseudo-measurements of the left and right feet, and the height pseudo-measurements of the left and right feet. The measurement covariance is then regulated as

(14) $$R_{t}=R_{\min}+\left(I_{14}-\Gamma_{t}\right)\left(R_{\max}-R_{\min}\right),$$

where $I_{14}$ is the 14×14 identity matrix, and $R_{t}$, $R_{\min}$, and $R_{\max}$ are diagonal positive-definite covariance matrices in $R^{14\times 14}$. The nominal covariance under reliable contact conditions is defined as $R_{0}=\mathrm{blkdiag}\left(R_{p,L}^{0},R_{p,R}^{0},R_{v,L}^{0},R_{v,R}^{0},R_{h,L}^{0},R_{h,R}^{0}\right)$, where $R_{p,i}^{0},R_{v,i}^{0}\in R^{3\times 3}$ are the diagonal reference position and velocity pseudo-measurement covariances of foot *i*, respectively, and $R_{h,i}^{0}>0$ is the corresponding nominal height variance. The covariance bounds are set as $R_{\min}=R_{0}$ and $R_{\max}=T_{R}R_{0}$. Consequently, the covariance blocks associated with each foot are continuously interpolated between the corresponding bounds, with lower contact reliability yielding larger measurement uncertainty. Since the same amplification factor $T_{R}$ is used in both the foot-position process covariance and the measurement covariance, their sensitivities to $T_{R}$ are $\partial Q_{i,t-1}^{f}/\partial T_{R}=\left(1-c_{i,t-1}\right)Q_{i,0}^{f}$ and $\partial R_{t}/\partial T_{R}=\left(I_{14}-\Gamma_{t}\right)R_{0}$, respectively. Thus, the effect of $T_{R}$ becomes stronger as the corresponding contact reliability decreases.

#### 2.4.3. Dynamic Residual Correction in Prediction

The dynamic residual compensates for time-varying base velocity prediction errors that remain after nominal state propagation and are not fully captured by the acceleration compensation state $b_{a,t}$ and the nominal process model.

Let $S_{x}={\left[\begin{array}{ccccc} 0_{3} & I_{3} & 0_{3} & 0_{3} & 0_{3} \end{array}\right]}^{T}\in R^{15\times 3}$ denote the velocity embedding matrix that maps the three-dimensional dynamic residual into the corresponding base velocity components of the filter state. The nominal and residual-corrected prior states are then given by

(15) $$\begin{array}{cc} {\hat{x}}_{t}^{-,0} & =A{\hat{x}}_{t-1}^{+}+Ba_{b,t-1}^{W},\\ {\hat{x}}_{t}^{-} & ={\hat{x}}_{t}^{-,0}+S_{x}r_{t}^{\mathrm{dyn}}. \end{array}$$

Here, $r_{t}^{\mathrm{dyn}}\in R^{3}$ is the dynamic residual in the base velocity space expressed in {W}. The terms ${\hat{x}}_{t-1}^{+}$, ${\hat{x}}_{t}^{-,0}$, and ${\hat{x}}_{t}^{-}$ denote the previous posterior estimate, nominal prior estimate, and residual-corrected prior estimate, respectively.

For covariance propagation, the residual-corrected prediction error is modeled as $x_{t}-{\hat{x}}_{t}^{-}=A\left(x_{t-1}-{\hat{x}}_{t-1}^{+}\right)+w_{t-1}+e_{t}^{\mathrm{res}}$, where $e_{t}^{\mathrm{res}}\in R^{15}$ denotes the remaining prediction error associated with the dynamic residual correction and is restricted to the base velocity components. This error is modeled as zero-mean with uncorrelated base velocity components. To account for the uncertainty associated with the learned correction, the variance of each affected component is modeled as proportional to the squared magnitude of the corresponding correction. Its covariance $Q_{t}^{\mathrm{res}}\in R^{15\times 15}$ is modeled as

(16) $$Q_{t}^{\mathrm{res}}=\mathrm{diag}\left[\kappa_{\mathrm{res}}{\left(S_{x}r_{t}^{\mathrm{dyn}}\right)}^{\circ 2}\right],$$

where diag(·) constructs a diagonal matrix from its vector argument, $\kappa_{\mathrm{res}}>0$ is the residual-uncertainty scaling coefficient, and ${\left(\cdot \right)}^{\circ 2}$ denotes elementwise squaring. For fixed dynamic residuals, the sensitivity to $\kappa_{\mathrm{res}}$ is $\partial Q_{t}^{\mathrm{res}}/\partial \kappa_{\mathrm{res}}=\mathrm{diag}\left[{\left(S_{x}r_{t}^{\mathrm{dyn}}\right)}^{\circ 2}\right]$. Thus, the sensitivity of each affected variance component is determined by the squared magnitude of the corresponding dynamic-residual component. For each nonzero component, the corresponding residual-related variance changes proportionally with $\kappa_{\mathrm{res}}$.

Assuming that $e_{t}^{\mathrm{res}}$ is uncorrelated with the previous posterior estimation error and the nominal process disturbance, the associated cross-covariance terms are neglected. The prediction covariance is therefore given by

(17) $$P_{t}^{-}=AP_{t-1}^{+}A^{T}+Q_{t-1}+Q_{t}^{\mathrm{res}}.$$

Here, $P_{t}^{-}$ and $P_{t-1}^{+}$ denote the prior and previous posterior estimation-error covariances, respectively. Since $S_{x}$ embeds the dynamic residual only into the base velocity components, $Q_{t}^{\mathrm{res}}$ affects only the corresponding base velocity covariance entries.

#### 2.4.4. Observation Residual Correction in Measurement Update

The observation residual is used to compensate for predictable pseudo-measurement errors that remain in the nominal kinematic observation model. Unlike the dynamic residual, which directly corrects the prior base velocity, the observation residual is defined in the measurement space and contributes to the state update by correcting the innovation. The residual-corrected innovation is defined as

(18) $$m_{t}=y_{t}-C{\hat{x}}_{t}^{-}-r_{t}^{\mathrm{obs}}.$$

Here, $r_{t}^{\mathrm{obs}}\in R^{14}$ denotes the observation residual used in the measurement update and represents the predictable additive error omitted from the nominal measurement model in (11).

Conditioned on the current input history, $r_{t}^{\mathrm{obs}}$ is treated as a deterministic estimate of the predictable component of the pseudo-measurement error. For the residual-corrected measurement update, the pseudo-measurement error is modeled as the sum of this predictable component and a remaining stochastic component, giving $y_{t}=Cx_{t}+r_{t}^{\mathrm{obs}}+e_{t}^{\mathrm{obs}}$, where $e_{t}^{\mathrm{obs}}\in R^{14}$ denotes the remaining measurement error after observation residual correction. This error is modeled as zero-mean with covariance $R_{t}$, where $R_{t}$ is the reliability-regulated covariance of the remaining pseudo-measurement error. Using the residual-corrected measurement model, the innovation becomes $m_{t}=C\left(x_{t}-{\hat{x}}_{t}^{-}\right)+e_{t}^{\mathrm{obs}}$. Assuming that the prior estimation error and $e_{t}^{\mathrm{obs}}$ are uncorrelated, the innovation covariance is $CP_{t}^{-}C^{T}+R_{t}$.

The uncertainty remaining after the observation residual correction is represented by $e_{t}^{\mathrm{obs}}$ and its reliability-regulated covariance $R_{t}$. Therefore, no additional residual-induced measurement covariance is introduced. The Kalman gain and posterior state estimate are therefore computed as

(19) $$\begin{array}{cc} K_{t} & =P_{t}^{-}C^{T}{\left(CP_{t}^{-}C^{T}+R_{t}\right)}^{-1},\\ {\hat{x}}_{t}^{+} & ={\hat{x}}_{t}^{-}+K_{t}m_{t}. \end{array}$$

For numerical robustness, the posterior covariance is updated in Joseph form as

(20) $$P_{t}^{+}=\left(I_{15}-K_{t}C\right)P_{t}^{-}{\left(I_{15}-K_{t}C\right)}^{T}+K_{t}R_{t}K_{t}^{T}.$$

#### 2.4.5. Estimation-Error Analysis

To examine the convergence and stability of the residual-corrected filtering process, define the posterior estimation error as $e_{t}^{+}=x_{t}-{\hat{x}}_{t}^{+}$. Combining the prediction-error model in Section 2.4.3 with the residual-corrected measurement update in Section 2.4.4 gives

(21) $$e_{t}^{+}=\left(I_{15}-K_{t}C\right)Ae_{t-1}^{+}+\left(I_{15}-K_{t}C\right)\left(w_{t-1}+e_{t}^{\mathrm{res}}\right)-K_{t}e_{t}^{\mathrm{obs}}.$$

Equation (21) separates the posterior estimation error into the propagation of the previous estimation error and the effects of the process disturbance and the remaining dynamic- and observation-residual errors. In the disturbance-free case, i.e., $w_{t-1}=0$, $e_{t}^{\mathrm{res}}=0$, and $e_{t}^{\mathrm{obs}}=0$, the error evolution is governed by the time-varying transition matrix $(I_{15}-K_{t}C)A$. If the error dynamics is uniformly exponentially stable on the contact-constrained observable subspace, the corresponding estimation error converges asymptotically to zero. In practice, persistent process and residual errors generally prevent exact convergence; if their combined effect has a bounded second moment, the posterior estimation error in this subspace remains mean-square bounded under the same stability condition. Global horizontal translation is not included in this convergence result because it is unobservable from the relative contact constraints without absolute position measurements.

### 2.5. Privileged Supervision and Multi-Task Training Objective

This subsection describes the privileged supervision signals and composite objective used to train the multi-task temporal estimator. Simulation-only contact information and reference states are used to supervise the contact reliability, dynamic residual, and observation residual, while online inference relies only on onboard proprioceptive measurements together with the motion command and nominal gait phase, without requiring foot-contact measurements or privileged simulation information.

#### 2.5.1. Privileged Contact Reliability Supervision

Privileged simulation information is used only during training to construct continuous supervision for the foot–ground kinematic constraint reliability branch. For foot i∈{L,R}, let $v_{i,t}^{W,\mathrm{gt}}\in R^{3}$ and $F_{i,t}^{W,\mathrm{gt}}\in R^{3}$ denote the foot linear velocity and resultant foot–ground contact force provided by the simulator and expressed in {W}, respectively. With $e_{z}={\left[0,0,1\right]}^{T}$ denoting the vertical unit vector expressed in {W}, the horizontal foot speed is defined as $v_{i,t}^{xy}={∥\left(I_{3}-e_{z}e_{z}^{T}\right)v_{i,t}^{W,\mathrm{gt}}∥}_{2}$, while the contact-force magnitude is given by $F_{i,t}={∥F_{i,t}^{W,\mathrm{gt}}∥}_{2}$. The horizontal-motion attenuation term, force-activation gate, and resulting continuous supervision target are defined as

(22) $$\begin{array}{cc} g_{i,t}^{v} & =\exp\;\left[-{\left(\frac{v_{i,t}^{xy}}{k_{v}}\right)}^{2}\right],\\ g_{i,t}^{F} & =\sigma \;\left(\frac{F_{i,t}-F_{\mathrm{th}}}{k_{F}}\right),\\ c_{i,t}^{\mathrm{tar}} & =g_{i,t}^{F}g_{i,t}^{v}. \end{array}$$

Here, σ(·) denotes the sigmoid function, $k_{v}>0$ is the horizontal-velocity scale, and $F_{\mathrm{th}}$ and $k_{F}>0$ are the contact-force threshold and force-gating scale, respectively. The product $c_{i,t}^{\mathrm{tar}}$ provides a continuous training surrogate for the validity of the stationary foot–ground kinematic constraint, rather than a binary contact label. It takes a high value only when the foot simultaneously carries sufficient contact force and exhibits small horizontal motion. Consequently, swing states with insufficient contact force are suppressed by $g_{i,t}^{F}$, whereas force-bearing contacts with appreciable horizontal motion are down-weighted by $g_{i,t}^{v}$.

#### 2.5.2. Multi-Task Training Objective

The composite training objective is defined as

(23) $$L=\lambda_{c}L_{c}+\lambda_{\mathrm{dyn}}L_{\mathrm{dyn}}+\lambda_{\mathrm{obs}}L_{\mathrm{obs}}+\lambda_{\mathrm{reg}}L_{\mathrm{reg}},$$

where $\lambda_{c}$, $\lambda_{\mathrm{dyn}}$, $\lambda_{\mathrm{obs}}$, and $\lambda_{\mathrm{reg}}$ denote the weighting coefficients for the contact reliability loss, dynamic-residual loss, observation-residual loss, and residual-magnitude regularization term, respectively.

The contact reliability loss directly supervises the continuous reliability generated after the gait-phase-constrained mapping:

(24) $$L_{c}=\frac{1}{|D_{c}|}\sum_{\left(i,t\right)\in D_{c}}{\left(c_{i,t}-c_{i,t}^{\mathrm{tar}}\right)}^{2},$$

where $D_{c}$ denotes the set of valid foot–time indices used for reliability supervision. This loss constrains the final continuous reliability output rather than directly supervising the latent variables, thereby preserving the structured gait-phase mapping.

Since the dynamic residual acts only in the base velocity space, its supervision loss is defined as

(25) $$L_{\mathrm{dyn}}=\frac{1}{|D_{v}|}\sum_{t\in D_{v}}{∥r_{t}^{\mathrm{dyn}}-\left(v_{b,t}^{W,\mathrm{gt}}-{\hat{v}}_{b,t}^{-,0}\right)∥}_{2}^{2},$$

where $D_{v}$ denotes the set of samples used for dynamic-residual supervision, $v_{b,t}^{W,\mathrm{gt}}$ denotes the simulator ground-truth base velocity expressed in {W}, and ${\hat{v}}_{b,t}^{-,0}$ denotes the base velocity component of the nominal prior estimate. The target corresponds to the discrepancy between the reference and nominal prior base velocities and therefore directly supervises the dynamic residual.

Let $x_{t}^{\mathrm{gt}}\in R^{15}$ denote a reference state arranged consistently with the filter state, with its physical state components constructed from simulator ground truth. The auxiliary acceleration-compensation component does not affect the observation-residual target because the corresponding columns of C are zero. The residual is applied to the 14 pseudo-measurement terms, and its supervision loss is formulated as

(26) $$L_{\mathrm{obs}}=\frac{1}{|D_{y}|}\sum_{t\in D_{y}}{∥r_{t}^{\mathrm{obs}}-\left(y_{t}-Cx_{t}^{\mathrm{gt}}\right)∥}_{2}^{2},$$

where $D_{y}$ denotes the set of samples used for measurement-space residual supervision. The loss guides the observation branch to compensate the residual error contained in the kinematic pseudo-measurements.

The residual magnitude is constrained by an additional regularization term:

(27) $$L_{\mathrm{reg}}=\frac{1}{\left|D\right|}\sum_{t\in D}\left[{∥r_{t}^{\mathrm{dyn}}∥}_{2}^{2}+{∥r_{t}^{\mathrm{obs}}∥}_{2}^{2}\right],$$

where D denotes the training sample set. This regularization term limits the magnitude of the dynamic and observation residuals and discourages excessive learned corrections from dominating the original model-based prediction and measurement processes.

## 3. Results

This section evaluates the proposed contact-reliability-aware neural residual Kalman filtering method through simulation and indoor real-robot experiments. The evaluation focuses on continuous contact reliability, dynamic and observation residual corrections, and overall state-estimation performance.

### 3.1. Simulation Setup

The simulation experiments were conducted using a self-developed bipedal robot model with 12 actuated joints. The robot has a height of 1.83m and a total mass of approximately 90kg. The multi-task temporal estimator was trained in an Isaac Lab environment based on NVIDIA Isaac Sim, whereas the state estimation experiments were performed in MuJoCo. Training was performed on a workstation equipped with an NVIDIA GeForce RTX 4090 GPU (24 GB VRAM). The training data were generated online from parallel rollouts of the pretrained locomotion policy in Isaac Lab. The motion commands were uniformly sampled within $v_{x}^{\mathrm{cmd}}\in \left[0,0.8\right]\;m/s$, $v_{y}^{\mathrm{cmd}}\in \left[-0.1,0.1\right]\;m/s$, and $\omega_{z}^{\mathrm{cmd}}\in \left[-0.3,0.3\right]\;\mathrm{rad}/s$. Training was conducted on random rough terrain with height perturbations in the range [−0.02,0.04]m, with a fixed random seed of 42. A total of 128 parallel environments were used, with 100 time steps collected from each environment per training iteration. The resulting samples were divided into 128 mini-batches of 100 temporal sequences, and the temporal estimator was optimized for two epochs per training iteration. Based on the approximate stabilization of the total training loss and the branch-specific losses, the training length was set to 3000 iterations, corresponding to approximately $3.84\times 10^{7}$ environment steps.

To ensure fair comparison, all methods were evaluated using the same locomotion policy, motion commands, proprioceptive inputs, and front-end attitude estimation method. The learning-based estimators used the same input configuration, history sequence length, Transformer backbone, and training settings, while the filter-based methods shared the same state-space model, initialization conditions, sampling period, and reference covariance parameters. In the corresponding ablation studies, only the contact reliability formulation or residual correction component under investigation was changed. The network architecture and training settings, continuous contact reliability parameters, and Kalman filter settings are summarized in Table 1, Table 2 and Table 3.

### 3.2. Evaluation Metrics

The state estimation accuracy is evaluated using the root mean square error (RMSE) of the base position, base velocity, and left and right foot positions, denoted by ${\mathrm{RM}}_{\mathrm{pos}}$, ${\mathrm{RM}}_{\mathrm{vel}}$, ${\mathrm{RM}}_{f,L}$, and ${\mathrm{RM}}_{f,R}$, respectively. For each three-dimensional state quantity, the componentwise RMSEs are first computed along the *x*-, *y*-, and *z*-axes, and their Euclidean norm is reported as the overall RMSE.

In addition, the terminal base position drift $e_{\mathrm{drift}}$ is used to quantify the accumulated position error at the end of a trajectory and is defined as the Euclidean distance between the estimated and reference base positions at the final time step. Since only the horizontal base position error is reported in the indoor experiments, the corresponding horizontal terminal drift is denoted by $e_{\mathrm{drift}}^{xy}$.

### 3.3. Comparison of Contact Reliability Modeling Strategies

To evaluate the influence of different contact reliability modeling strategies on state estimation performance, three reliability generation methods are compared within the same Kalman filtering framework. The Binary Contact Model generates binary contact reliability $c_{i,t}\in \left\{0,1\right\}$ using a threshold on the estimated foot height and switches the corresponding contact-related covariances accordingly. Here, the estimated foot height is taken as the vertical component of the foot-position estimate in the Kalman filter. The End-to-End Reliability Estimation method uses a Transformer to directly estimate continuous contact reliability $c_{i,t}\in \left[0,1\right]$ from historical input sequences. The Reliability-Aware Hybrid Model combines temporal estimation with the nominal gait phase and generates continuous contact reliability $c_{i,t}\in \left[0,1\right]$ through bounded gait-related parameters. The reliability values generated by all three methods regulate the same contact-related process and measurement covariances. Only the contact reliability generation strategy is varied, while all other experimental settings are kept unchanged.

The robot was commanded to walk on flat terrain at a forward velocity of 0.6m/s and a yaw angular velocity of 0.02rad/s, with a total test duration of 20s. For clear visualization of the temporal variations in contact reliability and estimation errors, Figure 3 shows only the data from 1 to 10s, whereas the quantitative metrics reported in Table 4 are computed over the complete 20s test sequence.

As shown in Figure 3, the three methods exhibit different temporal patterns of contact reliability. The Binary Contact Model switches only between 0 and 1 and therefore cannot represent the continuous variation in constraint reliability during contact transitions. Its position errors in the *x* and *y* directions increase noticeably over time. Although the End-to-End Reliability Estimation method produces continuous reliability values, noticeable decreases and fluctuations occur during parts of the support phase, while its horizontal position errors show the most pronounced growth. In comparison, the Reliability-Aware Hybrid Model produces higher reliability values during the support phase and smooth variations near touchdown and liftoff. Some fluctuations can still be observed during the non-support phase, but the corresponding horizontal position errors are smaller than those of the other two methods.

As summarized in Table 4, the Reliability-Aware Hybrid Model consistently outperforms the two reliability generation strategies. Relative to the Binary Contact Model, the reductions in ${\mathrm{RM}}_{\mathrm{pos}}$, ${\mathrm{RM}}_{\mathrm{vel}}$, and $e_{\mathrm{drift}}$ are approximately 57.8%, 32.3%, and 57.0%, respectively. Relative to the End-to-End Reliability Estimation method, the reductions increase to approximately 79.2%, 40.6%, and 78.7%. Overall, the Reliability-Aware Hybrid Model achieves the lowest estimation errors among the three strategies under the tested walking condition.

### 3.4. Ablation Study and Overall Estimation Performance

This subsection investigates the performance of the complete estimator and the contribution of each component through comparative and ablation studies. The Binary Contact Kalman filter (KF) serves as the baseline, where binary contact states generated by the Binary Contact Model are used to switch the contact-related covariances. The Continuous Contact KF replaces the binary strategy with the Reliability-Aware Hybrid Model and adjusts the corresponding covariances using continuous contact reliability. Starting from the Continuous Contact KF, the dynamic residual and observation residual are added individually, and their combined configuration forms the Proposed Pipeline, as listed in Table 5. Since continuous contact reliability and covariance regulation are jointly designed, they are treated as a single component in the ablation analysis. The main ablation experiments are performed on flat terrain, while additional tests on mildly uneven terrain are included to examine whether the observed estimation improvements persist under limited terrain variation.

The simulation evaluation was conducted on flat terrain, with additional tests on mildly uneven terrain. The commanded forward velocity and yaw angular velocity ranged from 0 to 0.8m/s and from −0.3 to 0.3rad/s, respectively. Ten trajectories were evaluated for each terrain condition, and each trajectory lasted 40s. For each state quantity, the RMSE along each axis was first calculated for every trajectory and then averaged across the 10 trajectories. The overall RMSE was obtained from the Euclidean norm of these three averaged componentwise RMSEs. A representative trajectory with a forward velocity of 0.6m/s and a yaw angular velocity of 0.02rad/s is selected for visualization, and the corresponding horizontal base trajectories are presented in Figure 4. The quantitative results are summarized in Table 6, including the RMSEs of the base position, base velocity, and left and right foot positions.

As illustrated in Figure 4, the horizontal trajectories obtained by the compared estimators show clear differences under the selected representative condition. The Continuous Contact KF decreases the horizontal terminal drift $e_{\mathrm{drift}}^{xy}$ from 1.992m and 1.766m to 0.806m and 0.802m on flat and mildly uneven terrains, respectively, compared with the Binary Contact KF. Adding the dynamic residual produces only limited changes in the horizontal trajectories, whereas adding the observation residual further reduces $e_{\mathrm{drift}}^{xy}$ to 0.111m and 0.427m on the two terrains, respectively.

Table 6 summarizes the quantitative results under both terrain conditions. Compared with the Binary Contact KF, the Continuous Contact KF reduces the base position, base velocity, and average foot-position RMSEs by approximately 61.0%, 32.2%, and 59.1% on flat terrain, respectively. Under mildly uneven terrain, the corresponding reductions are approximately 56.2%, 31.2%, and 54.7%. The average foot-position RMSE is calculated as the mean of the overall RMSEs of the left and right feet. Since the Continuous Contact KF incorporates continuous contact reliability together with covariance regulation, these results represent their combined contribution.

Based on the Continuous Contact KF, the dynamic and observation residuals affect different estimation metrics. With the dynamic residual enabled, the base-velocity RMSE decreases from 0.154m/s to 0.132m/s on flat terrain and from 0.159m/s to 0.149m/s on mildly uneven terrain, whereas the base position and foot-position errors show little variation. The observation residual mainly improves position-related estimates. On flat terrain, the base-position RMSE decreases from 0.465m to 0.065m, and the average foot-position RMSE decreases from approximately 0.501m to 0.107m. On mildly uneven terrain, the corresponding errors decrease from 0.476m and approximately 0.506m to 0.188m and 0.198m, respectively.

The Proposed Pipeline achieves base-position RMSEs of 0.065m and 0.188m on flat and mildly uneven terrains, respectively, with corresponding base-velocity RMSEs of 0.133m/s and 0.154m/s. Its base-position and foot-position errors are identical, at the reported precision, to those of the observation-residual variant under both terrain conditions, indicating that the position-related improvement is mainly associated with the observation residual. In contrast, the resulting base-velocity RMSE remains below that of the Continuous Contact KF but slightly above that of the dynamic-residual-only variant, indicating that the combined effect of the two residuals on the posterior base velocity is not a simple additive improvement.

### 3.5. Residual Contribution and Interaction Analysis

To examine the interaction between the dynamic and observation residuals within the recursive filtering process, their effects on the posterior base velocity are analyzed from both a representative walking segment and multiple test trajectories. For the representative segment, the contact reliability, dynamic residual, observation residual, and their respective contributions to the posterior base-velocity correction are recorded, as shown in Figure 5. This analysis extends the preceding ablation results by examining how the two residuals, although introduced at different stages of the filter, jointly affect the same posterior base-velocity subspace.

Figure 5a presents the continuous contact reliability of the left and right feet over the selected walking segment, reflecting the time-varying validity of the corresponding foot–ground kinematic constraints. In Figure 5b, the dynamic residual has a mean norm of $2.02\times 10^{-2}\;m/s$ and provides continuous compensation to the predicted base velocity at the prediction stage. Figure 5c presents the normalized $\ell_{2}$ norms of the observation residual components using fixed reference scales. The position component has the largest normalized magnitude, followed by the velocity component, whereas the height component remains smaller.

Because the dynamic and observation residuals act in different spaces, their original magnitudes cannot be directly compared. Their effects are therefore evaluated in the same posterior base-velocity subspace. The Dynamic correction in Figure 5d is defined as $\Delta v_{t}^{\mathrm{dyn}}=S_{x}^{T}\left(I_{15}-K_{t}C\right)S_{x}r_{t}^{\mathrm{dyn}}$, while the Observation correction is $\Delta v_{t}^{\mathrm{obs}}=-S_{x}^{T}K_{t}r_{t}^{\mathrm{obs}}$, where $S_{x}^{T}$ extracts the base-velocity components from the filter state. Net correction denotes $∥\Delta v_{t}^{\mathrm{dyn}}+\Delta v_{t}^{\mathrm{obs}}{∥}_{2}$, and Non-alignment denotes the gap between $∥\Delta v_{t}^{\mathrm{dyn}}{∥}_{2}+\;{∥\Delta v_{t}^{\mathrm{obs}}∥}_{2}$ and the Net correction. The mean correction norms associated with the Dynamic and Observation terms are $1.86\times 10^{-2}$ and $2.06\times 10^{-2}\;m/s$, respectively, showing comparable instantaneous correction magnitudes over the selected segment. These magnitudes describe their effects on the posterior base velocity rather than their respective improvements in estimation accuracy. As shown in Table 6, the reduction in base-velocity RMSE is mainly associated with the dynamic residual, whereas the observation residual mainly improves the base-position and foot-position estimates. For this representative segment, the mean Net correction is 79.3% of the mean sum of the Dynamic and Observation correction norms, indicating directional non-alignment between the two posterior corrections.

The posterior correction interaction was also evaluated across ten trajectories for each terrain condition. For trajectory *j*, the net-correction ratio was defined as

(28) $$\zeta_{j}=\frac{\sum_{t}{∥\Delta v_{j,t}^{\mathrm{dyn}}+\Delta v_{j,t}^{\mathrm{obs}}∥}_{2}}{\sum_{t}\left({∥\Delta v_{j,t}^{\mathrm{dyn}}∥}_{2}+{∥\Delta v_{j,t}^{\mathrm{obs}}∥}_{2}\right)},$$

and the directional alignment was defined as

(29) $$\xi_{j}=\frac{1}{|T_{j}|}\sum_{t\in T_{j}}\frac{{\left(\Delta v_{j,t}^{\mathrm{dyn}}\right)}^{T}\Delta v_{j,t}^{\mathrm{obs}}}{{∥\Delta v_{j,t}^{\mathrm{dyn}}∥}_{2}{∥\Delta v_{j,t}^{\mathrm{obs}}∥}_{2}},$$

where $T_{j}$ denotes the time steps at which both posterior corrections have nonzero magnitudes. Across the ten trajectories, the mean ± standard deviation of $\zeta_{j}$ and $\xi_{j}$ were 0.781±0.009 and 0.135±0.035 on flat terrain, respectively, and 0.783±0.006 and 0.105±0.034 on mildly uneven terrain. The sub-unity ζ and small positive ξ show that the two posterior base-velocity corrections are not fully aligned and exhibit only weak average directional alignment. Their effects in the shared posterior base-velocity subspace are therefore coupled and non-additive. The ablation results are consistent with this interaction. The dynamic residual provides the main base-velocity improvement, whereas the observation residual primarily improves the base-position and foot-position estimates while also modifying the posterior base velocity through the Kalman measurement update. The Proposed Pipeline maintains a lower aggregate base-velocity RMSE than the Continuous Contact KF, while its value remains slightly higher than that of the dynamic-residual-only variant under both terrain conditions.

### 3.6. Indoor Experiments

The indoor experiments assess the trajectory estimation accuracy and onboard execution of the proposed estimator using real robot sensing data. A motion-capture system was used to obtain the reference base motion. Four reflective markers attached to the robot trunk formed a rigid cluster, allowing the base position to be reconstructed at a sampling rate of 120Hz. The horizontal speed derived from the processed motion-capture trajectory was used to describe the actual locomotion process. During the experiments, both the Binary Contact KF and the Proposed Pipeline received onboard IMU and joint encoder measurements without using foot-contact measurements. The proposed estimator was deployed on an NVIDIA Jetson AGX Orin onboard computer running Ubuntu 20.04. The temporal estimator was converted to Open Neural Network Exchange (ONNX) format and executed as part of the onboard estimation pipeline. The temporal-estimator inference required an average of 14.32ms, while the subsequent continuous contact reliability generation, forward kinematics, translational Jacobian computation, and Kalman filtering required an average of 12.14ms, resulting in an average end-to-end latency of 26.46ms. The estimator operated with an update period of approximately 50ms. The measured maximum end-to-end latency of the complete pipeline was 36.24ms, which was within the scheduled update interval for the physical experiments.

Estimation performance was evaluated through a system-level comparison between the Binary Contact KF and the Proposed Pipeline. During the experiments, locomotion commands were manually issued using a handheld controller. The commanded forward velocity was gradually increased from rest to approximately 0.35m/s, followed by steady walking and subsequent deceleration to rest, while an additional yaw rate command was applied in the turning test. The motion-capture data were temporally synchronized with the estimator outputs, and the initial horizontal positions were aligned to remove the constant origin offset. Figure 6 shows the physical robot and motion-capture setup, while Figure 7 presents representative data from the first 30s of the two experiments. Figure 7a,b show the horizontal base trajectories for the straight walking and turning tests, respectively. Figure 7c,d show the corresponding horizontal position errors, while Figure 7e,f show the horizontal speed profiles derived from the motion-capture trajectories. The speed profiles are used to characterize the executed locomotion process over the displayed time interval, whereas the horizontal position errors reflect the corresponding trajectory estimation performance. The quantitative metrics computed over the complete experimental trajectories are summarized in Table 7.

Compared with the Binary Contact KF, the Proposed Pipeline reduces both the horizontal base-position RMSE and terminal drift in the straight-walking and turning tests, with RMSE reductions of 31.3–53.9% and terminal-drift reductions of 71.6–75.5%. The larger reduction in terminal drift indicates a smaller accumulated horizontal position error at the end of each test. After normalization by the motion-capture reference path length, the horizontal position RMSE of the Proposed Pipeline corresponds to only 2.43–2.68% of the reference path length. Together with the horizontal trajectories and position error profiles in Figure 7, these results show that the Proposed Pipeline produces horizontal base trajectories closer to the motion-capture reference under the tested straight walking and turning conditions. Since the physical experiments evaluate the complete estimation pipeline at the system level, the observed improvements are interpreted as the combined effect of the integrated estimator rather than being attributed to any individual contact reliability or residual module.

## 4. Discussion

In legged robot state estimation without foot-contact measurements, binary contact states cannot fully reflect the instantaneous validity of foot–ground kinematic constraints, whereas continuous contact reliability allows the confidence assigned to these constraints to vary with the contact condition. Nominal state propagation and foot kinematic pseudo-measurements also contain motion-dependent errors that cannot be addressed by contact-related covariance regulation alone. The proposed method therefore uses continuous contact reliability to regulate the contact-related covariances, while the dynamic and observation residuals correct the prior base velocity and measurement innovation, respectively. The different improvements observed in the ablation study are consistent with these distinct roles in the Kalman recursion.

In the contact reliability comparison, the end-to-end method produces continuous contact reliability, although noticeable variations remain during some stance intervals. Lower reliability enlarges the corresponding process and measurement covariances, reducing the weight assigned to otherwise valid foot–ground kinematic constraints. The Reliability-Aware Hybrid Model instead uses the nominal gait phase as a structural prior and permits bounded adjustments of phase offset, swing ratio, and touchdown/liftoff transition parameters. This preserves the expected contact timing structure while allowing limited timing deviations, thereby reducing unnecessary reliability variations during stance.

The ablation results show distinct effects of the two residuals. The dynamic residual directly corrects the prior base velocity and mainly reduces velocity error, with limited changes in the base and foot position estimates. The observation residual modifies the foot kinematic pseudo-measurement innovation and affects the state through the Kalman gain, producing larger improvements in base and foot position estimation. In contrast to CoCo-InEKF and related covariance-learning methods, the proposed estimator uses structured continuous reliability for contact-related uncertainty regulation and applies separate residual corrections during prediction and measurement update. The full model does not minimize every metric. Its base-velocity RMSE is slightly higher than that of the dynamic-residual-only variant, while the position accuracy of the observation-residual variant is retained and the velocity error remains below that of the Continuous Contact KF. The two residuals therefore provide complementary corrections at different stages of the Kalman recursion.

In the physical-robot experiments, the complete framework runs online using IMU and encoder measurements within the onboard computational budget. It also yields lower horizontal position errors and terminal drift than the Binary Contact KF during straight walking and turning. The proposed framework is not restricted to the specific bipedal platform used in this study. It can be extended to other bipedal and quadrupedal robots, after adapting the state-space model, leg kinematics, input dimensions, and covariance settings to the target platform. The contact-reliability-aware covariance regulation and stage-specific residual correction mechanisms can retain their basic form after these platform-specific adaptations. The learned temporal estimator, however, may require fine-tuning or retraining when robot morphology, sensor configuration, gait pattern, or operating conditions differ from those used for training. Beyond single-robot deployment across different platforms, the proposed framework could also be applied to multi-robot cooperative localization, with an adapted estimator running locally on each robot. Inter-robot relative pose observations can provide additional constraints on the local proprioceptive estimates, limiting the growth of relative-pose errors and thereby reducing the relative drift that would otherwise accumulate when the robots estimate their states independently.

The main limitations are associated with the gait-phase prior, the data dependence of the temporal estimator, and the current validation scope. The contact-reliability model relies on a nominal gait phase and assumes that the actual touchdown and liftoff times do not deviate excessively from the nominal gait schedule. Severe slip, strong disturbances, or nonperiodic stepping may shift touchdown or liftoff timing beyond the prescribed adjustment range, while operation far outside the training distribution may also degrade estimation performance. The current physical-robot experiments are limited to indoor level-ground walking and turning, and more complex contact conditions and real uneven terrain have not yet been evaluated. Without absolute position observations, the estimator can reduce but cannot eliminate long-term global position drift. Visual, light detection and ranging (LiDAR), or other localization information could therefore be incorporated for long-duration operation.

## 5. Conclusions

In legged robot state estimation without foot-contact measurements, this paper addresses estimation errors associated with variations in the validity of foot–ground kinematic constraints and residual errors in state propagation and foot kinematic pseudo-measurements. The proposed contact-reliability-aware neural residual Kalman filtering framework uses a shared temporal representation of proprioceptive history to jointly generate continuous contact reliability, a dynamic residual, and an observation residual. Contact reliability regulates the contact-related covariances, while the two residuals correct the prior base velocity during prediction and the measurement innovation during update. In this way, the explicit state-space model and recursive Kalman structure are retained, with structured contact reliability modeling and stage-specific residual compensation incorporated into the filtering process. Simulation results show improved estimation of the base and foot states. Physical-robot experiments further demonstrate more accurate horizontal base trajectories and lower terminal drift during straight walking and turning. The onboard implementation meets the prescribed online update requirement.

Future work will investigate more flexible contact-timing adaptation and improve the generalization and transferability of the temporal estimator across operating conditions and robot platforms. Physical-robot validation will also be extended to more complex terrains and contact scenarios, together with the integration of exteroceptive localization for long-duration state estimation.

## Figures and Tables

**Figure 1:**
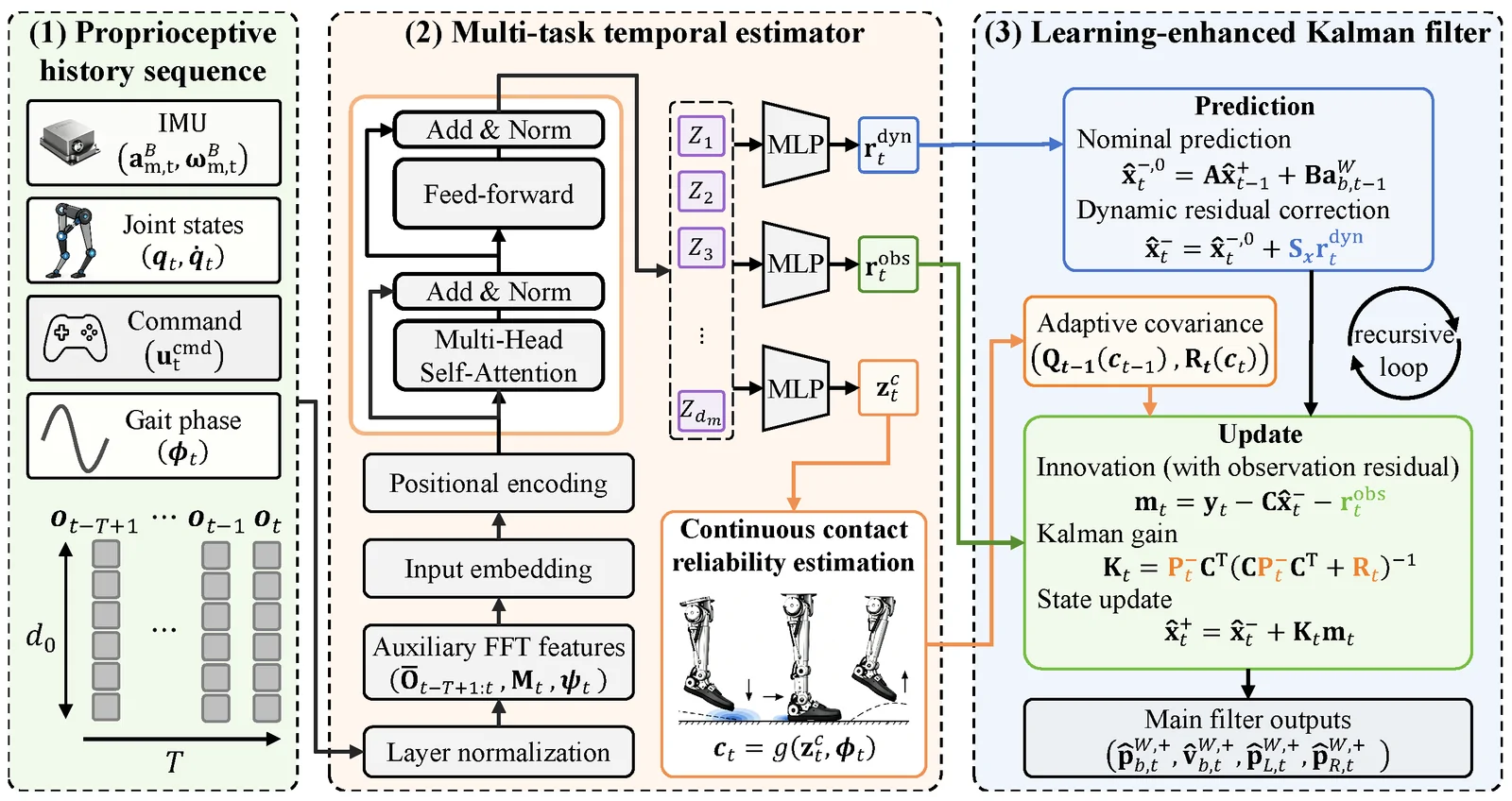
Overall architecture of the proposed contact-reliability-aware neural residual Kalman filtering framework. Black, blue, green, and orange arrows denote the main information flow, dynamic residual flow, observation residual flow, and contact-reliability-driven covariance regulation flow, respectively.

**Figure 2:**
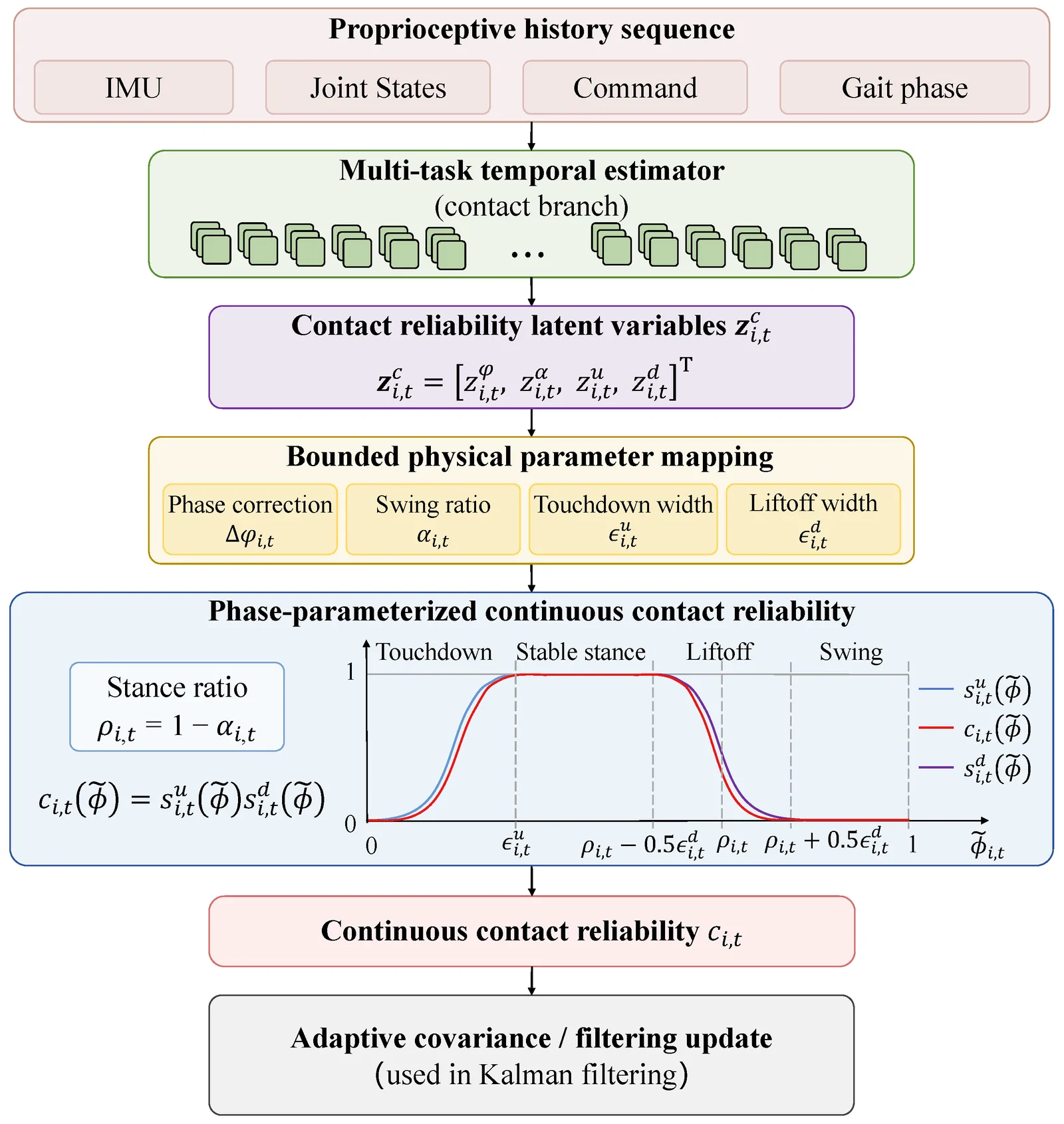
Generation of continuous contact reliability from the nominal gait phase and contact-related latent variables. The latent variables provide bounded corrections to the gait-phase parameters and transition widths used to construct the continuous reliability profile.

**Figure 3:**
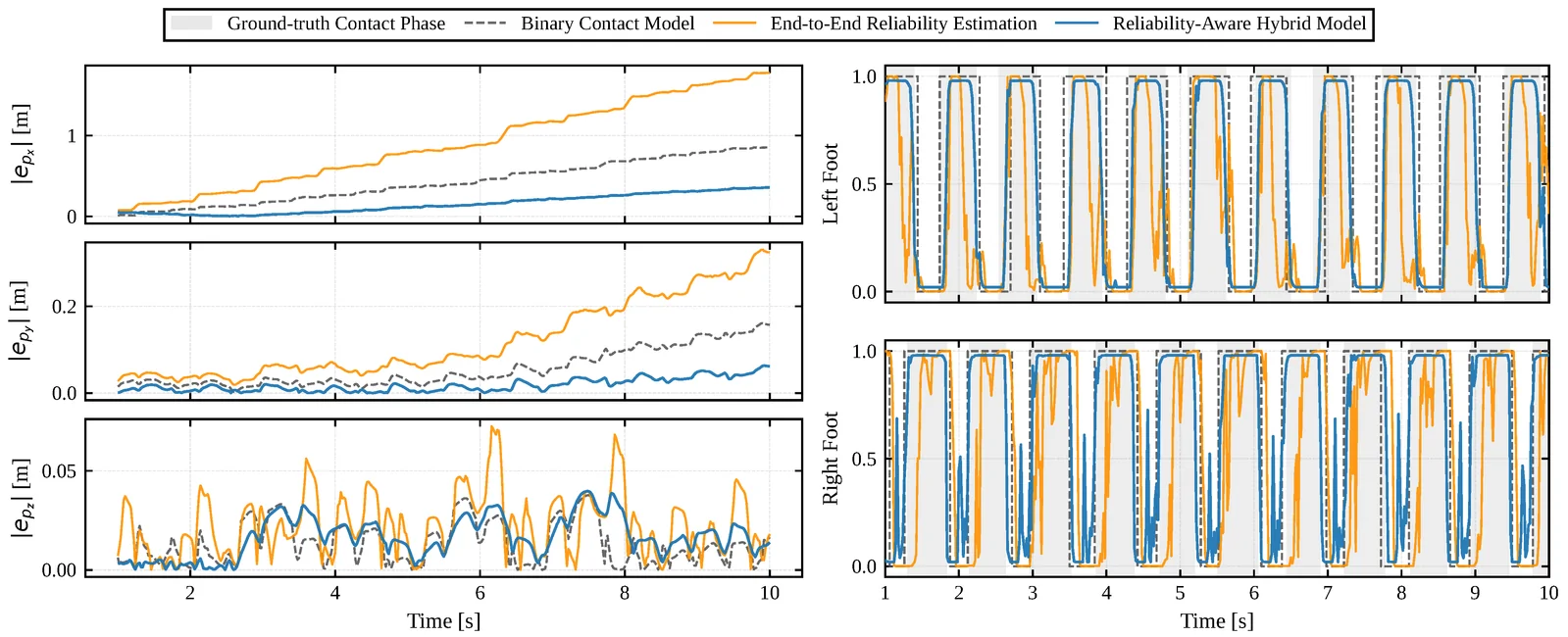
Comparison of contact reliability modeling strategies in simulation. The left panels show the base position errors along the *x*-, *y*-, and *z*-axes, while the right panels show the contact reliability of the left and right feet. Gray shaded regions indicate the ground-truth contact phases.

**Figure 4:**
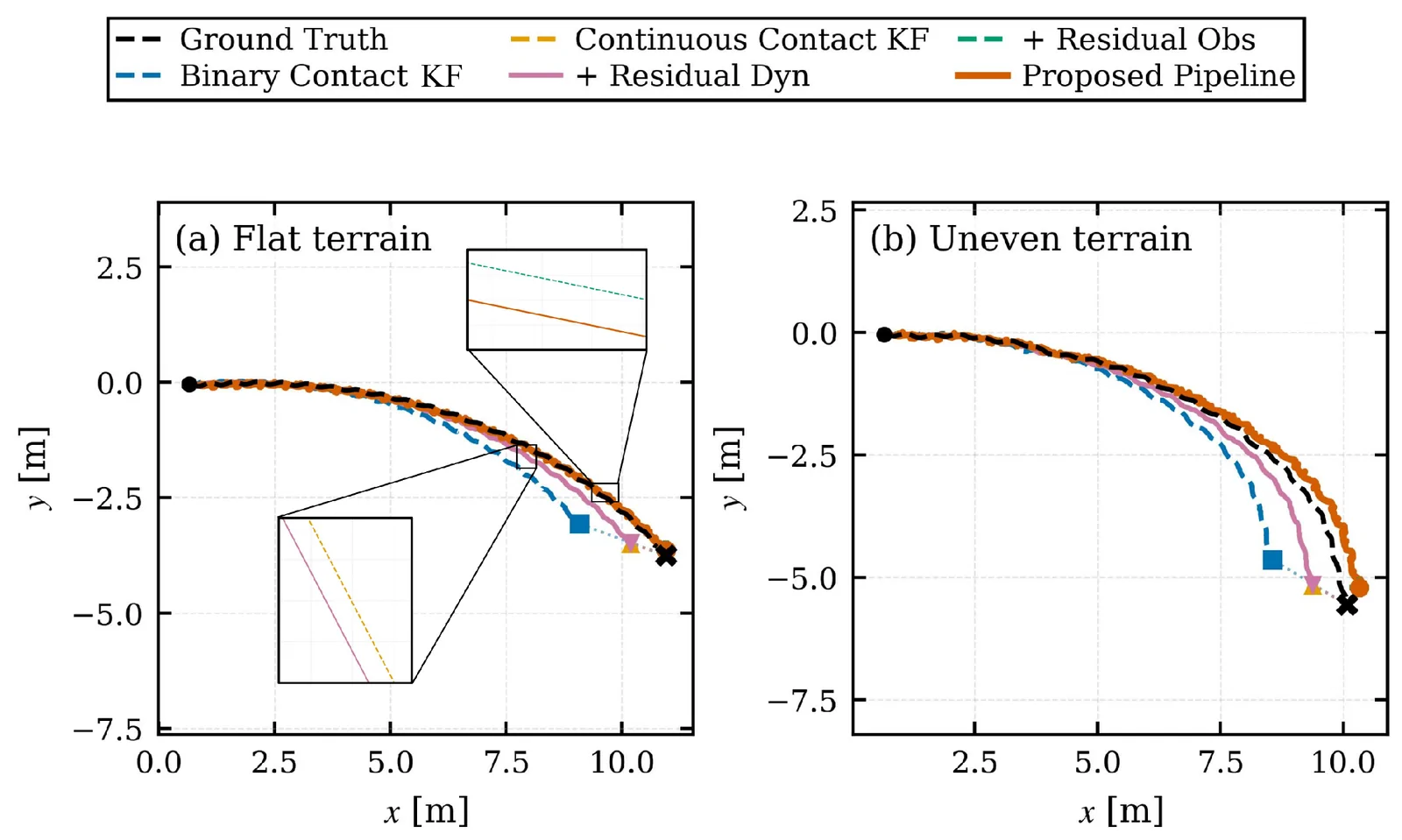
Comparison of horizontal base trajectories on flat and mildly uneven terrains. The left and right panels correspond to flat and mildly uneven terrain, respectively, and compare the estimated *x*–*y* trajectories of the different filtering variants with the simulation ground truth.

**Figure 5:**
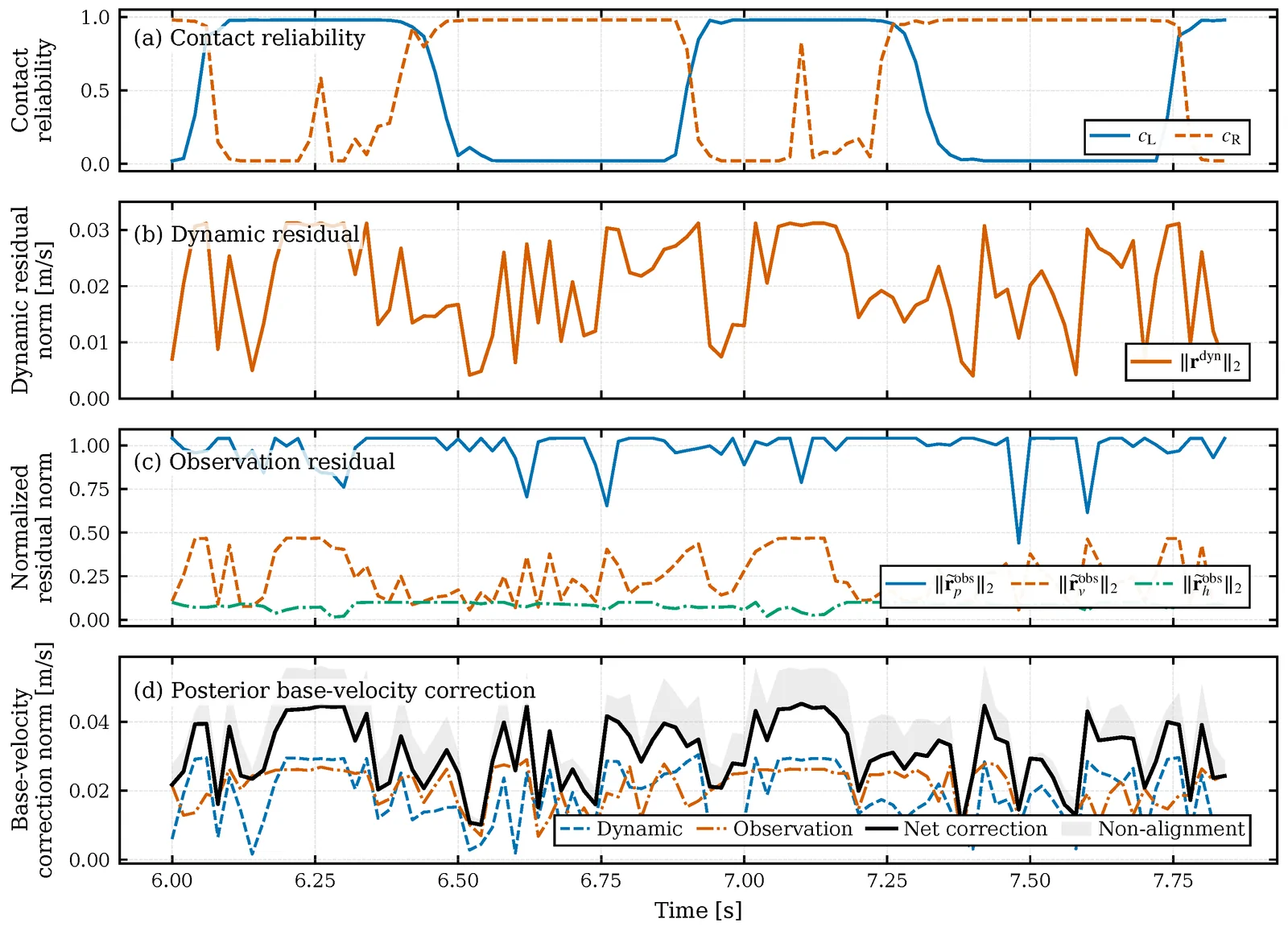
Residual evolution and posterior correction contributions during a representative walking segment. (**a**) Continuous contact reliability of the left and right feet, $c_{L}$ and $c_{R}$. (**b**) $\ell_{2}$ norm of the dynamic residual $r^{\mathrm{dyn}}$ in the base-velocity space. (**c**) Grouped $\ell_{2}$ norms of the normalized observation residual. ${\tilde{r}}_{p}^{\mathrm{obs}}$, ${\tilde{r}}_{v}^{\mathrm{obs}}$, and ${\tilde{r}}_{h}^{\mathrm{obs}}$ are the normalized position, velocity, and height pseudo-measurement residuals. (**d**) Posterior base-velocity corrections associated with the dynamic and observation residuals. Dynamic and Observation show their respective correction magnitudes, Net correction shows the magnitude of their vector sum, and the shaded region indicates directional non-alignment.

**Figure 6:**
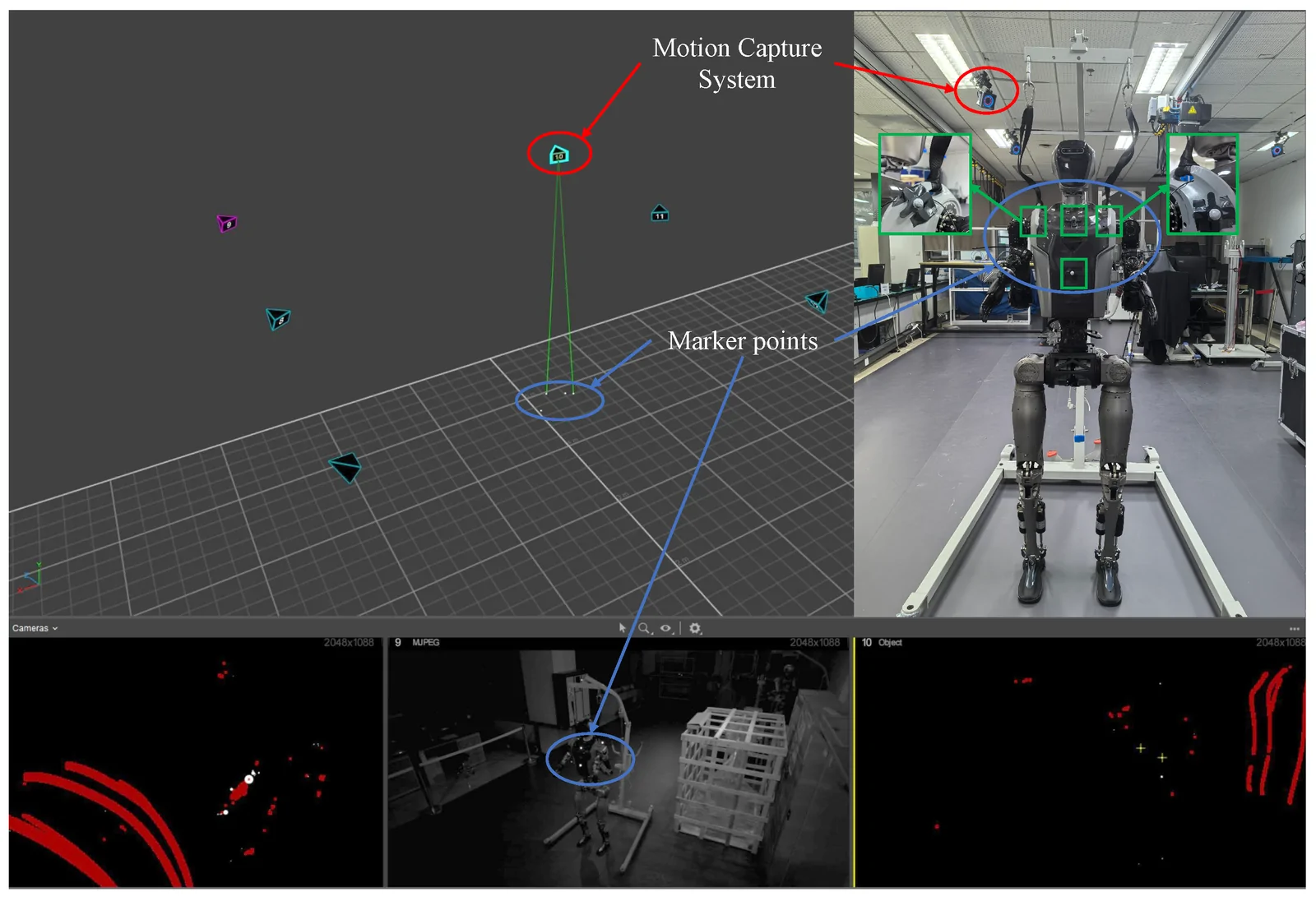
Indoor motion-capture setup and marker-tracking visualization. Four reflective markers attached to the robot trunk form a rigid cluster for reconstructing the reference base position.

**Figure 7:**
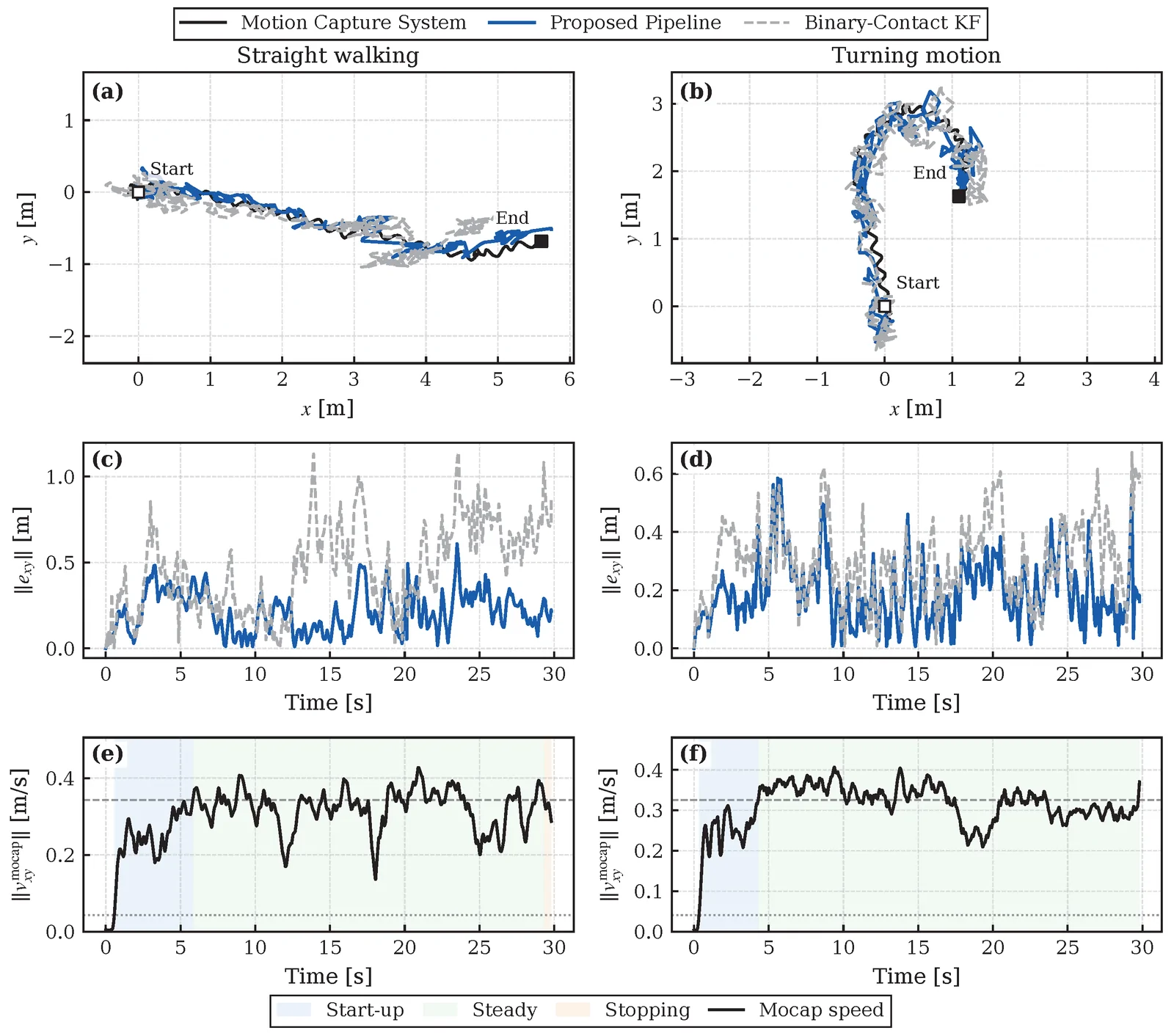
Indoor motion-capture evaluation under straight walking and turning conditions. (**a**) Horizontal base trajectory in the straight walking test. (**b**) Horizontal base trajectory in the turning test. (**c**) Horizontal base-position error in the straight walking test. (**d**) Horizontal base-position error in the turning test. (**e**) Motion-capture-derived horizontal speed profile in the straight walking test. (**f**) Motion-capture-derived horizontal speed profile in the turning test.

**Table 1:** Architecture and training settings of the multi-task temporal estimator.

Parameter	Value	Description
Temporal Estimator Architecture		
*T*	50	History length
$N_{h}$	16	Attention heads
$N_{e}$	4	Encoder layers
$p_{d}$	0.1	Dropout rate
$d_{m}$	128	Model dimension
$d_{\mathrm{ff}}$	512	Feed-forward dimension
$d_{e}$	128	Input embedding dimension
$d_{c}$	32	Contact branch dimension
$d_{\mathrm{dyn}}$	[128,64]	Dynamic branch sizes
$d_{\mathrm{obs}}$	[64,32]	Observation branch sizes
Training Configuration		
Optimizer	Adam	Optimization algorithm
η	$1.0\times 10^{-4}$	Learning rate
$\lambda_{c}$	1.0	Contact loss weight
$\lambda_{\mathrm{dyn}}$	0.1	Dynamic loss weight
$\lambda_{\mathrm{obs}}$	0.1	Observation loss weight
$\lambda_{\mathrm{reg}}$	0.001	Regularization weight

**Table 2:** Parameters for continuous contact reliability generation and privileged supervision.

Parameter	Value	Description
Contact Reliability Generation		
$\Delta \varphi_{i}^{0}$	−0.28	Nominal phase bias
$\Delta \varphi_{\max}$	0.20	Maximum phase correction
$\alpha_{i}^{0}$	0.38	Nominal swing ratio
$\Delta \alpha_{\max}$	0.15	Maximum swing-ratio correction
$\left[\alpha_{\min},\alpha_{\max}\right]$	[0.15,0.65]	Swing-ratio bounds
$\epsilon_{\min}$	0.02	Minimum transition width
$\epsilon_{\mathrm{range}}$	0.10	Transition-width range
β	0.45	Transition constraint factor
γ	8.0	Transition sharpness
$\left[c_{\min},c_{\max}\right]$	[0.02,0.98]	Reliability clipping range
Supervision Label Construction		
$k_{v}$	0.07m/s	Horizontal-velocity scale
$F_{\mathrm{th}}$	50.0N	Contact-force threshold
$k_{F}$	10.0N	Force-gating scale

**Table 3:** Kalman filter settings and covariance parameters.

Parameter	Value	Description
Process Covariance		
$Q_{p}$	$\mathrm{diag}(5.0\;\times \;10^{-3},\;5.0\;\times \;10^{-3},\;5.0\;\times \;10^{-4})$	Base Position
$Q_{v}$	$\mathrm{diag}(1.0\;\times \;10^{-5},\;2.0\;\times \;10^{-7},\;1.0\;\times \;10^{-4})$	Base velocity
$Q_{i,0}^{f}$	$\mathrm{diag}(1.0\;\times \;10^{-6},\;1.0\;\times \;10^{-6},\;1.0\;\times \;10^{-6})$	Foot position
$Q_{b_{a}}$	$\mathrm{diag}(1.0\;\times \;10^{-6},\;1.0\;\times \;10^{-6},\;1.0\;\times \;10^{-8})$	Acceleration compensation
$Q_{a}$	$\mathrm{diag}(1.0\;\times \;10^{-4},\;1.0\;\times \;10^{-4},\;4.0\;\times \;10^{-4})$	Acceleration input
Measurement Covariance		
$R_{p,L}^{0}$	$\mathrm{diag}(1.0\;\times \;10^{-8},1.0\;\times \;10^{-8},5.0\;\times \;10^{-11})$	Left-foot position
$R_{p,R}^{0}$	$\mathrm{diag}(6.0\;\times \;10^{-7},8.0\;\times \;10^{-8},1.0\;\times \;10^{-11})$	Right-foot position
$R_{v,L}^{0}$	$\mathrm{diag}(1.0\;\times \;10^{-4},2.0\;\times \;10^{-4},1.0\;\times \;10^{-5})$	Left-foot velocity
$R_{v,R}^{0}$	$\mathrm{diag}(5.0\;\times \;10^{-5},1.0\;\times \;10^{-4},5.0\;\times \;10^{-6})$	Right-foot velocity
$R_{h,L}^{0}$	$5.0\;\times \;10^{-4}$	Left-foot height
$R_{h,R}^{0}$	$5.0\;\times \;10^{-4}$	Right-foot height
Filter Settings and Scaling Parameters		
$P_{0}$	$0.1\;I_{15}$	Initial covariance
$T_{R}$	100.0	Covariance amplification factor
$\kappa_{\mathrm{res}}$	$1.0\;\times \;10^{-3}$	Residual covariance scale

**Table 4:** Comparison of state estimation errors under different contact reliability modeling strategies.

Method	${\mathrm{RM}}_{\mathrm{pos}}$ [m]	${\mathrm{RM}}_{\mathrm{vel}}$ [m/s]	$e_{\mathrm{drift}}$ [m]
Binary Contact Model	1.122	0.229	1.906
End-to-End Reliability Estimation	2.271	0.261	3.852
Reliability-Aware Hybrid Model	0.473	0.155	0.819

**Table 5:** Ablation settings of the compared estimators.

Method	Contact Reliability	Dynamic Residual	Observation Residual
Binary Contact KF	Binary	×	×
Continuous Contact KF	Continuous	×	×
+Dynamic Residual	Continuous	✓	×
+Observation Residual	Continuous	×	✓
Proposed Pipeline	Continuous	✓	✓

Note: ✓ and × indicate enabled and disabled residual components, respectively.

**Table 6:** Quantitative comparison results on flat and mildly uneven terrains.

Terrain	Metric	Binary Contact KF	Continuous Contact KF	+Dynamic Residual	+Observation Residual	Proposed Pipeline
Flat	${\mathrm{RM}}_{\mathrm{pos}}$ [m]	1.155 0.287 0.008 (1.191)	0.452 0.108 0.005 (0.465)	0.452 0.108 0.005 (0.465)	0.039 0.052 0.004 (0.065)	0.039 0.052 0.004 (0.065)
	${\mathrm{RM}}_{\mathrm{vel}}$ [m/s]	0.131 0.136 0.125 (0.227)	0.062 0.126 0.065 (0.154)	0.062 0.096 0.065 (0.132)	0.064 0.121 0.067 (0.152)	0.063 0.097 0.067 (0.133)
	${\mathrm{RM}}_{f,L}$ [m]	1.182 0.303 0.083 (1.224)	0.478 0.124 0.075 (0.500)	0.478 0.124 0.075 (0.500)	0.038 0.056 0.079 (0.104)	0.038 0.056 0.079 (0.104)
	${\mathrm{RM}}_{f,R}$ [m]	1.184 0.303 0.083 (1.225)	0.480 0.124 0.075 (0.502)	0.480 0.123 0.075 (0.502)	0.044 0.061 0.079 (0.109)	0.044 0.061 0.079 (0.109)
Uneven Terrain	${\mathrm{RM}}_{\mathrm{pos}}$ [m]	1.009 0.402 0.021 (1.087)	0.443 0.170 0.024 (0.476)	0.443 0.170 0.024 (0.476)	0.138 0.127 0.021 (0.188)	0.138 0.127 0.021 (0.188)
	${\mathrm{RM}}_{\mathrm{vel}}$ [m/s]	0.122 0.137 0.140 (0.231)	0.078 0.122 0.065 (0.159)	0.075 0.110 0.065 (0.149)	0.083 0.117 0.069 (0.159)	0.079 0.113 0.069 (0.154)
	${\mathrm{RM}}_{f,L}$ [m]	1.030 0.424 0.065 (1.116)	0.463 0.192 0.057 (0.505)	0.463 0.192 0.057 (0.505)	0.118 0.146 0.060 (0.197)	0.118 0.146 0.060 (0.197)
	${\mathrm{RM}}_{f,R}$ [m]	1.033 0.423 0.065 (1.118)	0.466 0.192 0.057 (0.507)	0.466 0.192 0.057 (0.507)	0.118 0.148 0.060 (0.199)	0.118 0.148 0.060 (0.199)

Note: Each entry reports the componentwise RMSEs along the *x*, *y*, and *z* axes averaged over the 10 test trajectories, followed by the overall RMSE in parentheses, computed as the Euclidean norm of the three averaged componentwise RMSEs.

**Table 7:** Quantitative comparison in indoor motion-capture experiments.

Test	Method	$L_{\mathrm{xy}}$ [m]	${\mathrm{RM}}_{\mathrm{pos}}^{\mathrm{xy}}$ [m] (%)	$e_{\mathrm{drift}}^{\mathrm{xy}}$ [m] (%)
Straight walking	Binary Contact KF	9.102	0.529 (5.81)	0.883 (9.70)
	Proposed Pipeline	9.102	0.244 (2.68)	0.216 (2.37)
	Reduction	–	53.9%	75.5%
Turning motion	Binary Contact KF	9.236	0.326 (3.53)	0.566 (6.13)
	Proposed Pipeline	9.236	0.224 (2.43)	0.161 (1.74)
	Reduction	–	31.3%	71.6%

Note: $L_{xy}$ denotes the horizontal reference path length. ${\mathrm{RM}}_{\mathrm{pos}}^{xy}$ and $e_{\mathrm{drift}}^{xy}$ denote the horizontal base-position RMSE and terminal drift, respectively. Parenthesized values are normalized by $L_{xy}$, and reductions are relative to the Binary Contact KF.

## Data Availability

The data that support the findings of this study are available from the corresponding author upon reasonable request.

## Appendix A. Summary of Principal Symbols and Notation

For reference, the principal symbols and notation used throughout the manuscript are summarized in Table A1.

**Table A1:** Summary of the principal symbols and notation used in this work.

Symbol	Description	Symbol	Description
*t*	Filter time index	$x_{t}$	Filter state
i∈{L,R}	Foot index	${\hat{x}}_{t}^{-},{\hat{x}}_{t}^{+}$	Prior and posterior state estimates
{B},{W}	Body and world frames	$p_{b,t}^{W}$	Base position in the world frame
$R_{B,t}^{W}$	Body-to-world rotation matrix	$v_{b,t}^{W}$	Base linear velocity in the world frame
$o_{t}$	Proprioceptive input vector	$p_{L,t}^{W},p_{R,t}^{W}$	Left- and right-foot positions
$O_{t-T+1:t}$	Proprioceptive history sequence	$b_{a,t}$	Acceleration-compensation state
$a_{m,t}^{B}$	IMU specific-force measurement	A,B	State-transition and acceleration-input matrices
$\omega_{m,t}^{B}$	IMU angular-velocity measurement	C	Measurement matrix
$q_{t},{\dot{q}}_{t}$	Joint-position and joint-velocity vectors	$y_{t}$	Contact-aided pseudo-measurement vector
$u_{t}^{\mathrm{cmd}}$	Motion command vector	$m_{t}$	Residual-corrected innovation
$\phi_{t}$	Nominal gait-phase vector	$Q_{t-1}$	Process covariance
$f_{\theta }\left(\cdot \right)$	Multi-task temporal estimator	$R_{t}$	Reliability-regulated measurement covariance
$z_{t}^{c}$	Contact-related latent representation	$\Gamma_{t}$	Contact-reliability matrix
$z_{i,t}^{c}$	Per-foot contact-related latent vector	$P_{t}^{-},P_{t}^{+}$	Prior and posterior error covariances
$e_{t}^{+}$	Posterior estimation error	$e_{t}^{\mathrm{res}},e_{t}^{\mathrm{obs}}$	Remaining prediction and measurement errors
$r_{t}^{\mathrm{dyn}}$	Dynamic residual	$S_{x}$	Velocity embedding matrix
$r_{t}^{\mathrm{obs}}$	Observation residual	$K_{t}$	Kalman gain
$\Delta v_{t}^{\mathrm{dyn}}$	Dynamic-residual posterior base-velocity correction	$\Delta v_{t}^{\mathrm{obs}}$	Observation-residual posterior base-velocity correction
$\zeta_{j}$	Net-correction ratio for trajectory *j*	$\xi_{j}$	Directional alignment for trajectory *j*
${\tilde{\phi }}_{i,t}$	Corrected gait phase	$c_{i,t}^{\mathrm{tar}}$	Contact-reliability supervision target
$\alpha_{i,t},\rho_{i,t}$	Swing and stance phase ratios	${\left(\cdot \right)}^{\mathrm{gt}}$	Privileged simulation ground-truth quantity
$\epsilon_{i,t}^{u},\epsilon_{i,t}^{d}$	Touchdown and liftoff transition widths	$L_{c},L_{\mathrm{dyn}}$	Contact-reliability and dynamic-residual losses
$s_{i,t}^{u},s_{i,t}^{d}$	Touchdown rising and liftoff falling functions	$L_{\mathrm{obs}},L_{\mathrm{reg}}$	Observation-residual and regularization losses
$c_{i,t}$	Continuous contact reliability of foot *i*	L	Composite training objective
$c_{t}$	Left- and right-foot reliability vector	Δt	Filter sampling period

## References

[1] Yang S., Zhang Z., Bokser B., Manchester Z. Multi-IMU proprioceptive odometry for legged robots. Proceedings of the 2023 IEEE/RSJ International Conference on Intelligent Robots and Systems (IROS).

[2] Ma H., Song A., Li J., Ge L., Fu C., Zhang G. (2025). Legged odometry based on fusion of leg kinematics and IMU information in a humanoid robot. Biomim. Intell. Robot..

[3] Bloesch M., Hutter M., Hoepflinger M.A., Leutenegger S., Gehring C., Remy C.D., Siegwart R. State estimation for legged robots: Consistent fusion of leg kinematics and IMU. Proceedings of the Robotics: Science and Systems.

[4] Hartley R., Ghaffari M., Eustice R.M., Grizzle J.W. (2020). Contact-aided invariant extended Kalman filtering for robot state estimation. Int. J. Robot. Res..

[5] Agrawal V., Bertrand S., Griffin R.J., Dellaert F. Proprioceptive state estimation of legged robots with kinematic chain modeling. Proceedings of the 2022 IEEE-RAS 21st International Conference on Humanoid Robots (Humanoids).

[6] Xavier F.E., Burger G., Pétriaux M., Deschaud J.-E., Goulette F. Multi-IMU proprioceptive state estimator for humanoid robots. Proceedings of the 2023 IEEE/RSJ International Conference on Intelligent Robots and Systems (IROS).

[7] Margolis G.B., Agrawal P. (2023). Walk These Ways: Tuning Robot Control for Generalization with Multiplicity of Behavior. Proceedings of the 6th Conference on Robot Learning, Auckland, New Zealand, 14–18 December 2022.

[8] Griffin R.J., McCrory S., Bertrand S., Calvert D., Lee I., Neuhaus P.D., Stephen D., Jasper J., Karumanchi S., Kourchians A. (2022). Quadrupedal walking over complex terrain with a quasi-direct drive actuated robot. Field Robot..

[9] Roychoudhury A., Khorshidi S., Agrawal S., Bennewitz M. (2023). Perception for humanoid robots. Curr. Robot. Rep..

[10] Tariq O., Han D. (2024). 2D Particle Filter Accelerator for Mobile Robot Indoor Localization and Pose Estimation. IEEE Access.

[11] Camurri M., Fallon M., Bazeille S., Radulescu A., Barasuol V., Caldwell D.G., Semini C. (2017). Probabilistic contact estimation and impact detection for state estimation of quadruped robots. IEEE Robot. Autom. Lett..

[12] Menner M., Berntorp K. Simultaneous state estimation and contact detection for legged robots by multiple-model Kalman filtering. Proceedings of the 2024 European Control Conference (ECC).

[13] Maravgakis M., Argiropoulos D.-E., Piperakis S., Trahanias P. Probabilistic contact state estimation for legged robots using inertial information. Proceedings of the 2023 IEEE International Conference on Robotics and Automation (ICRA).

[14] Liu W., Sun S., Liu H., Song W. State estimation for point-foot parallel-legged bipedal robot. Proceedings of the 2024 43rd Chinese Control Conference (CCC).

[15] Kim J.-H., Hong S., Ji G., Jeon S., Hwangbo J., Oh J.-H., Park H.-W. (2021). Legged robot state estimation with dynamic contact event information. IEEE Robot. Autom. Lett..

[16] Yoon Z., Kim J.-H., Park H.-W. (2024). Invariant smoother for legged robot state estimation with dynamic contact event information. IEEE Trans. Robot..

[17] Chen B., Zang X., Song C., Zhang Y., Zhang X., Zhao J. (2025). Noise analysis and hierarchical adaptive body state estimator for biped robot walking with ESVC foot. IEEE Sens. J..

[18] Kim K.-H., Ahn D., Lee D.-H., Yoon J., Hyun D.J. Adaptive invariant extended Kalman filter for legged robot state estimation. Proceedings of the 2025 IEEE/RSJ International Conference on Intelligent Robots and Systems (IROS).

[19] Wan M., Liu D., Wu J., Li L., Peng Z., Liu Z. (2024). State estimation for quadruped robots on non-stationary terrain via invariant extended Kalman filter and disturbance observer. Sensors.

[20] Baumgartner M., Müller D., Serifi A., Grandia R., Knoop E., Gross M., Bächer M. CoCo-InEKF: State estimation with learned contact covariances in dynamic, contact-rich scenarios. Proceedings of the Robotics: Science and Systems.

[21] Buchanan R., Camurri M., Dellaert F., Fallon M. (2022). Learning inertial odometry for dynamic legged robot state estimation. Proceedings of the 5th Conference on Robot Learning, London, UK, 8–11 November 2021.

[22] Yu C., Yang Y., Liu T., You Y., Zhou M., Xiang D. State estimation transformers for agile legged locomotion. Proceedings of the 2024 IEEE/RSJ International Conference on Intelligent Robots and Systems (IROS).

[23] Wasserman J., Agarwal A., Jangir R., Chowdhary G., Pathak D., Gupta A. (2025). Legolas: Deep Leg-Inertial Odometry. Proceedings of the 8th Conference on Robot Learning, Munich, Germany, 6–9 November 2024.

[24] Yazdipaz K., Kohli N., Golestaneh S.A., Shahbazi M. (2025). Robust and efficient phase estimation in legged robots via signal imaging and deep neural networks. IEEE Access.

[25] Lin T.-Y., Zhang R., Yu J., Ghaffari M. (2022). Legged robot state estimation using invariant Kalman filtering and learned contact events. Proceedings of the 5th Conference on Robot Learning, London, UK, 8–11 November 2021.

[26] Youm D., Oh H., Choi S., Kim H., Jeon S., Hwangbo J. Legged robot state estimation with invariant extended Kalman filter using neural measurement network. Proceedings of the 2025 IEEE International Conference on Robotics and Automation (ICRA).

[27] Liu W., Caruso D., Ilg E., Dong J., Mourikis A.I., Daniilidis K., Kumar V., Engel J. (2020). TLIO: Tight learned inertial odometry. IEEE Robot. Autom. Lett..

[28] Liu X., Bao Y., Cheng P., Shen D., Fan Z., Xu H., Chen G. (2025). Learning-assisted multi-IMU proprioceptive state estimation for quadruped robots. Information.

[29] Hohmeyer L., Popescu M., Bergonzani I., Mronga D., Kirchner F. InEKFormer: A hybrid state estimator for humanoid robots. Proceedings of the 2025 IEEE International Conference on Advanced Robotics (ICAR).

[30] Gu Y., Liu Z., Zou T. Enhancing leg odometry in legged robots with learned contact bias: An LSTM recurrent neural network approach. Proceedings of the 2024 IEEE/RSJ International Conference on Intelligent Robots and Systems (IROS).

[31] Lee S., Kim H.-B., Kim K.-S. Legged robot state estimation using invariant neural-augmented Kalman filter with a neural compensator. Proceedings of the 2025 IEEE/RSJ International Conference on Intelligent Robots and Systems (IROS).

[32] Lee S., Kim K.-S. (2026). Attention-based neural-augmented Kalman filter for legged robot state estimation. IEEE Robot. Autom. Lett..

[33] Zhou T., Ma Z., Wen Q., Wang X., Sun L., Jin R. (2022). FEDformer: Frequency enhanced decomposed transformer for long-term series forecasting. Proceedings of the 39th International Conference on Machine Learning, Baltimore, MD, USA, 17–23 July 2022.

[34] Vaswani A., Shazeer N., Parmar N., Uszkoreit J., Jones L., Gomez A.N., Kaiser L., Polosukhin I. (2017). Attention is all you need. Advances in Neural Information Processing Systems.

